# Oxygen-Coordinated Single Mn Sites for Efficient Electrocatalytic Nitrate Reduction to Ammonia

**DOI:** 10.1007/s40820-023-01217-z

**Published:** 2023-11-06

**Authors:** Shengbo Zhang, Yuankang Zha, Yixing Ye, Ke Li, Yue Lin, Lirong Zheng, Guozhong Wang, Yunxia Zhang, Huajie Yin, Tongfei Shi, Haimin Zhang

**Affiliations:** 1 Laboratory of Materials Physics, Centre for Environmental and Energy Nanomaterials, Anhui Key Laboratory of Nanomaterials and Nanotechnology, CAS Center for Excellence in Nanoscience, Institute of Solid State Physics, HFIPS, Chinese Academy of Sciences, Hefei, 230031 People’s Republic of China; 2 University of Science and Technology of China, Hefei, 230026 People’s Republic of China; 3 Key Laboratory of Agricultural Sensors, Ministry of Agriculture, School of Information and Computer, Anhui Agricultural University, Hefei, 230026 People’s Republic of China; 4 Hefei National Research Center for Physical Sciences at the Microscale, University of Science and Technology of China, Hefei, 230026 People’s Republic of China; 5 Beijing Synchrotron Radiation Facility, Institute of High Energy Physics, Chinese Academy of Sciences, 19B Yuquan Road, Beijing, 100049 People’s Republic of China

**Keywords:** Atomically dispersed, Oxygen coordination, Nitrate reduction reaction, In situ spectroscopic studies, Hydrogen evolution reaction

## Abstract

Electrocatalytic nitrate reduction reaction has attracted increasing attention due to its goal of low carbon emission and environmental protection. Here, we report an efficient NitRR catalyst composed of single Mn sites with atomically dispersed oxygen (O) coordination on bacterial cellulose-converted graphitic carbon (Mn–O–C). Evidence of the atomically dispersed Mn–(O–C_2_)_4_ moieties embedding in the exposed basal plane of carbon surface is confirmed by X-ray absorption spectroscopy. As a result, the as-synthesized Mn–O–C catalyst exhibits superior NitRR activity with an NH_3_ yield rate (R_NH3_) of 1476.9 ± 62.6 μg h^**−**1^ cm^**−**2^ at − 0.7 V (vs. reversible hydrogen electrode, RHE) and a faradaic efficiency (FE) of 89.0 ± 3.8% at − 0.5 V (vs. RHE) under ambient conditions. Further, when evaluated with a practical flow cell, Mn–O–C shows a high R_NH3_ of 3706.7 ± 552.0 μg h^**−**1^ cm^**−**2^ at a current density of 100 mA cm^**−**2^, 2.5 times of that in the H cell. The in situ FT-IR and Raman spectroscopic studies combined with theoretical calculations indicate that the Mn–(O–C_2_)_4_ sites not only effectively inhibit the competitive hydrogen evolution reaction, but also greatly promote the adsorption and activation of nitrate (NO_3_^−^), thus boosting both the FE and selectivity of NH_3_ over Mn–(O–C_2_)_4_ sites.

**Supplementary Information:**

The online version contains supplementary material available at 10.1007/s40820-023-01217-z.

## Highlights

- Oxygen-coordinated single-atom Mn catalyst was fabricated via introducing oxygen functional groups rich bacterial cellulose as the adsorption regulator through a combined impregnation–pyrolysis–etching synthetic route.
- Mn–O–C as the electrocatalyst exhibits superior electrocatalytic activity toward ammonia synthesis with a maximum NH_3_ yield rate of 1476.9 ± 62.6 μg h^**−**1^ cm^**−**2^ at − 0.7 V (vs. RHE) and a faradaic efficiency of 89.0 ± 3.8% at − 0.5 V (vs. RHE) under ambient conditions.
- Electrocatalytic mechanism of Mn–(O–C_2_)_4_ site for nitrate reduction reaction is unveiled by a combination of in situ spectroscopy characterization and computational study.

**Supplementary Information:**

The online version contains supplementary material available at 10.1007/s40820-023-01217-z.

## Graphical abstract

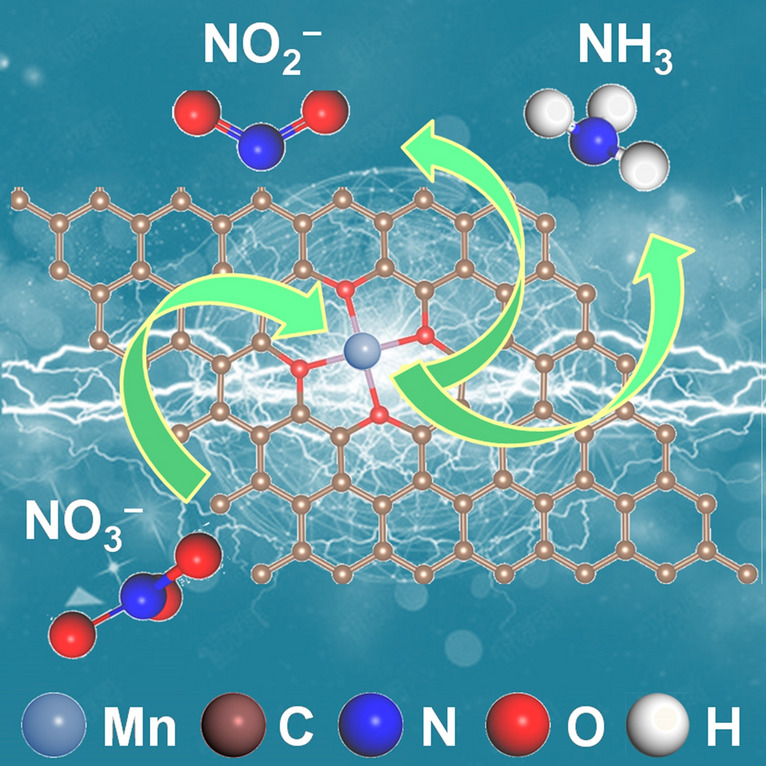

## Introduction

Ammonia (NH_3_) is an important large-scale industrial product in the fertilizer industry, which has attracted widespread attention as one of the most promising low-carbon energy carriers with low liquefaction pressure and high hydrogen density [1–8]. Industrially, NH_3_ synthesis is still dominated by the long-standing Harber–Bosch process under harsh conditions, which consumes approximately 1.4% of annual energy consumption and approximately 3% of global carbon dioxide (CO_2_) emissions [9–12]. Electrocatalytic N_2_ reduction reaction (NRR) is generally considered an energy-efficient and sustainable process for synthesizing NH_3_ at ambient conditions [13, 14]. However, the NH_3_ selectivity (S_NH3_) and yield rate (R_NH3_) are greatly hindered by the high dissociation energy of N≡N tripe bond (941 kJ mol^−1^), poor solubility of N_2_ in the electrolyte and the competitive hydrogen evolution reaction (HER) [15, 16]. Compared with NRR, the electrocatalytic nitrate reduction reaction (NitRR) to NH_3_ is not limited by the low solubility of N_2_ in aqueous environment, and its thermodynamics is more advantageous because the dissociation energy of the N = O bond (204 kJ mol^−1^) is much lower than the N≡N tripe bond (941 kJ mol^−1^) [17, 18]. It is worth noting that the nitrate (NO_3_^−^) widely exists in industrial and agricultural wastewater, posing a real potential threat to human health and natural balance [19–23]. Therefore, converting nitrate into valuable and recyclable NH_3_ is a frontier field that requires in-depth research. However, in the NitRR process, the competitive HER and the complex eight-electron reduction processes hinder the faradaic efficiency (FE) and selectivity of NH_3_ [24–27]. Thus, there is an urgent need for efficient NitRR catalysts with both activity and selectivity simultaneously.

Previous studies have reported that due to the appropriate energy and symmetric 3d orbitals of Mn, the Mn–O site has the ability to catalyze NRR, which is beneficial for the adsorption and activation of N_2_ molecules [28, 29]. In addition, benefiting from the minimum metal sizes, atomically dispersed single-atom catalysts (SACs) are expected to have great potential to improve and may therefore convert NO_3_^−^ into NH_3_ at an acceptable overpotential. Meanwhile, the uniform active sites within atomically dispersed metal catalysts ensure high selectivity, thus ensuring a satisfactory FE [30–33].

Inspired by the above breakthroughs and our previous research on NitRR and SACs [34–37], we have rationally modulated oxygen (O)-coordinated single-atom Mn catalyst (Mn–O–C) with Mn–(O–C_2_)_4_ as a novel NitRR active coordination configuration. The bacterial cellulose (BC) with rich oxygen functional groups is innovatively utilized as Mn^2+^ impregnation regulator and the precursor to simultaneously derive the carbon support (CBC) and anchor Mn single atoms to CBC via Mn–O coordination bonds during a facile carbothermal reduction process. The resultant Mn–O–C achieves a high FE of 89.0 ± 3.8% at − 0.5 V (vs. RHE) and a desirable R_NH3_ of 1476.9 ± 62.6 μg h^**−**1^ cm^**−**2^ at − 0.7 V (vs. RHE) under ambient conditions. Our density functional theory (DFT) calculations reveal that introduction of Mn–(O–C_2_)_4_ sites renders NO_3_^−^ chemisorption, activates the hydrogenation of adsorbed NO_3_^−^ and suppresses HER, ultimately enhancing the selectivity of NitRR.

## Experimental Section

### Preparation of Mn NPs/CBC and Mn–O-C

BC pellicle was frozen by liquid nitrogen and freeze-dried in a bulk tray dryer at a sublimating temperature of − 75 °C and a pressure of 0.01 mbar for 48 h. The pre-treated BC was used as the adsorbent to controllably impregnate Mn^2+^. The BC (1.0 g) was immersed in a 400-mL solution containing 16 mmol of MnSO_4_·4H_2_O (concentration of Mn^2+^: 40 mmol L^**−**1^) for 6.0 h. The BC with adsorbed Mn^2+^ was adequately washed with deionized water, freeze-dried and subjected to the pyrolytic treatment at 360 °C for 2.0 h and then 700 °C for 3.0 h under Ar atmosphere to carbothermally reduce the adsorbed Mn^2+^ on BC to metallic Mn NPs and simultaneously carbonize BC into graphitic carbon (CBC). The resultant Mn NPs/CBC was adequately washed with deionized water and ethanol and dried at 60 °C under vacuum for 6.0 h, followed by a refluxing acid-etching process using 2.0 M H_2_SO_4_ at 120 °C for 6.0 h to remove metallic Mn NPs. The resultant Mn–O–C was adequately washed with distilled water and ethanol and dried under vacuum for 12 h.

### Electrochemical Measurements

The electrochemical measurements were carried out in a customized H-type glass cell separated by Nafion 211 membrane at room temperature. A CHI 760E electrochemical workstation (CH Instrumental Corporation, Shanghai, China) was used to record the electrochemical response. The Mn–O–C sample on carbon paper, saturated Ag/AgCl electrode and platinum mesh was used as the working electrode, reference electrode and counter electrode, respectively. Before use, the Nafion 211 membrane was successively treated at 80 °C in H_2_O_2_ (5.0 wt%) and 0.1 M H_2_SO_4_ aqueous solutions and thoroughly rinsed with the deionized water. The working electrode was prepared as follows: 2.5 mg of the targeted electrocatalyst was firstly dispersed in 950 µL of absolute ethanol and 50 µL of Nafion solution (5.0 wt%) under sonication for 30 min to form a homogeneous ink. One hundred microliters of ink was loaded onto a carbon paper (1.0 × 1.0 cm^2^) and dried under ambient conditions for 40 min before use. The surface area of carbon paper was 0.25 mg cm^**−**2^ as the working electrode. The as-fabricated electrode was treated in Ar-saturated 0.1 M K_2_SO_4_ + 1000 ppm N-KNO_3_ solution to activate before being used. It should be noted that the reported NH_3_ yield in this work is the NH_3_ product collected only from cathodic compartment. In this work, all measured potentials versus Ag/AgCl were converted to the potentials versus RHE (*E*_RHE_) according to the following equation:

1 $$ E_{{{\text{RHE}}}} = E_{{{\text{Ag}}/{\text{AgCl}}}} + 0.059{\text{pH}} + E_{{{\text{Ag}}/{\text{AgCl}}}}^{ \circ } $$

where *E*_Ag/AgCl_ is the equilibrium potential under standard conditions and *E*^o^_Ag/AgCl_ = 0.1967 V versus RHE at 25 °C.

The commercial gas diffusion electrode (GDE) consisted of a working electrode, Nafion 211 membrane and platinum foil anode. The effective catalytic area was 1.0 cm^2^ with a catalyst loading of 0.25 mg cm^**−**2^. Using Ar-saturated 0.1 M K_2_SO_4_ + 1000 ppm N-KNO_3_ as both the flowing cathode and anode electrolyte, the synthesized NH_3_ can be transported out at a flexible rate. The electrocatalytic nitrate reduction reaction was tested at the constant current density of 50, 100 and 150 mA cm^**−**2^. Each experiment was run in triplicate, and the average values with error bars are presented.

The yielded ammonia and the content of nitrite in the electrolyte were measured by colorimetric methods [34, 36, 37].

### In situ Raman and FT-IR Spectroscopy Measurements

For the in situ Raman spectroscopy tests, the samples were recorded on a RXN1-785 Raman spectrometer (Analytik Jena AG, excited wavelength of 785 nm) connected with the CHI 660E electrochemical workstation. The in situ attenuated total reflection surface-enhanced infrared adsorption spectroscopy (ATR-SEIRAS) was performed on a FT-IR spectrometer (Nicolet iS50, Thermo Scientific) equipped with an MCT-A detector with silicon as the prismatic window. Fist, Mn–O–C ink (pure ethanol as a dispersant) was carefully dropped on the surface of gold film, which was chemically deposited on the surface of the silicon prismatic before each experiment. Then, the deposited silicon prismatic served as the working electrode. The platinum mesh and Ag/AgCl electrode containing saturated KCl solution were used as the counter and reference electrodes, respectively. The 0.1 M K_2_SO_4_ + 1000 ppm N-KNO_3_ solution was employed as the electrolyte. Each infrared absorption spectrum was acquired by averaging 128 scans at a resolution of 4.0 cm^−1^. The background spectrum of the catalyst electrode was acquired at an open-circuit voltage before each systemic measurement, and the measured potential ranges of the electrocatalytic NitRR were − 0.2 to − 0.7 V versus RHE with an interval of 0.1 V.

## Results and Discussion

### Synthesis and Characterizations of Mn–O–C

**Fig. 1:**
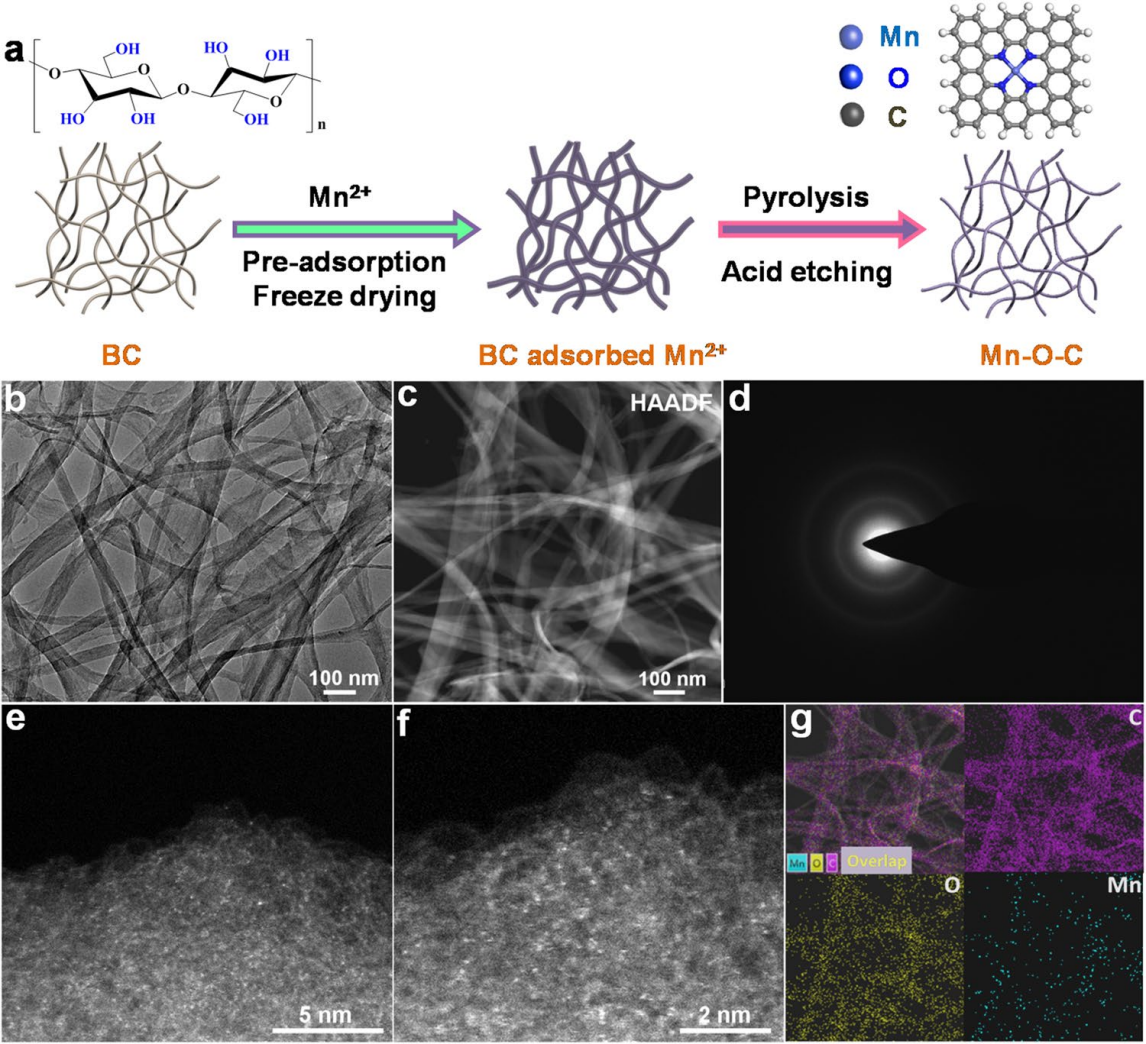
**a** Schematic for diagram illustrating synthetic procedure of Mn–O–C. **b** TEM image, **c** HAADF-STEM image, and **d** SAED pattern of Mn–O–C. **e, f** Enlarged aberration-corrected HAADF-STEM images of Mn–O–C. **g** Elemental mapping of Mn–O–C

Similar to the synthetic method we previously reported [34–36], bacterial cellulose (BC) with rich O-containing functional groups and nanofiber network structures (Fig. S1) was used as the adsorption regulator to controllably adsorb Mn^2+^, followed by freeze-drying, high-temperature pyrolysis and acid washing treatment, to achieve oxygen-coordinated single Mn sites with certain loading supported on BC-converted graphitic carbon (Mn–O–C) (Fig. 1a). As revealed by the transmission electron microscopy (TEM), the as-prepared Mn–O–C still maintains the initial fiber-like aggregation morphology after carbonization fixation and acid-etching process (Fig. 1b). Further observations by high-angle annular dark-field scanning transmission electron microscopy (HAADF-STEM) indicate that no metal nanoparticles had formed (Fig. 1c). The dominant diffraction peaks corresponding to carbon in the X-ray diffraction (XRD) pattern together with the broad D and G bonds in the Raman spectrum (Fig. S2) are consistent with a partially graphitized carbon structures, which is confirmed by the selected area electron diffraction (SAED) image (Fig. 1d). The enlarged aberration-corrected HAADF-STEM images of Mn–O–C confirm that the Mn atoms were atomically dispersed on the CBC support (Fig. 1e, f). Homogeneously dispersed single Mn sites can clearly be observed according to the different intensity profiles (Fig. S3). In addition, the elemental mappings reveal the homogenous distribution of C, O and Mn over the whole CBC support (Fig. 1g). The actual loading of Mn in Mn–O–C sample was measured to be 1.01% by the inductively coupled plasma atomic emission spectrometer (ICP-AES) analysis. The N_2_ physisorption isotherm and pore size distribution demonstrate its high Brunauer–Emmett–Teller (BET) specific surface area of 496.8 m^2^ g^−1^ and micro- and mesoporous structure (Fig. S4), which are beneficial to exposure of the isolated Mn sites and the mass transport of electrolytes during electrolysis [34–36].

### Confirmation of Mn–(O–C_2_)_4_ Single-atom Site

**Fig. 2:**
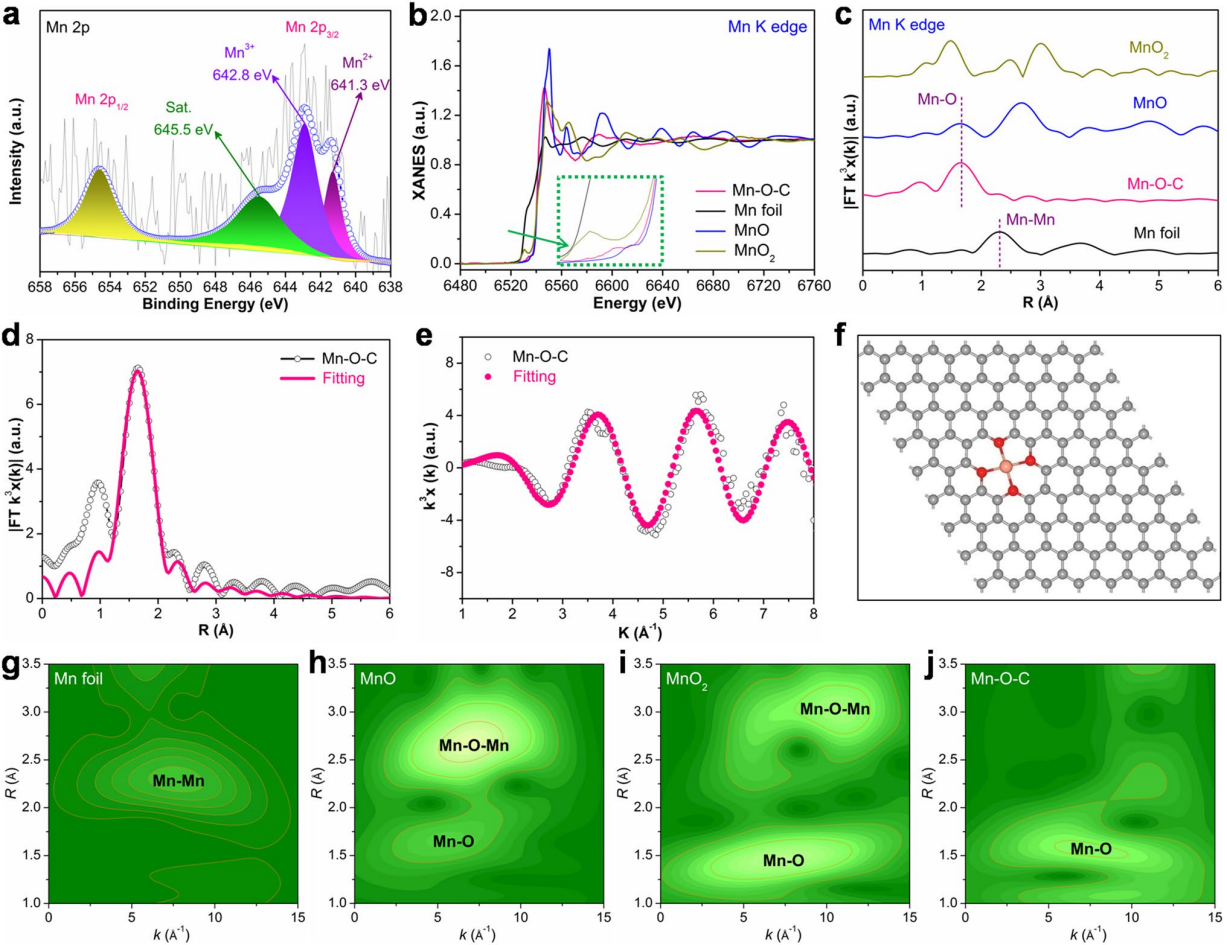
**a** High-resolution XPS spectra of Mn 2*p* of Mn–O–C. **b** Mn *K* edge XANES spectra and **c** *k*^3^-weighted FT-EXAFS spectra of Mn–O–C and references. **d**, **e** Mn *K* edge EXAFS fitting curves of Mn–O–C at *R* space and *k* space. **f** The proposed Mn–(O–C_2_)_4_ coordination configuration in Mn–O–C (C, O and Mn atoms are in color of gray, red and orange, respectively). **g–j** Mn *K* edge WT-EXAFS of Mn–O–C and references

X-ray photoelectron spectroscopy (XPS) was then used to characterize the Mn–O–C catalyst surface and composition (Fig. S5). As shown in Fig. S5a, the survey XPS spectra show the presence of Mn, O and C elements in the Mn–O–C sample. The corresponding elements contents show that the atomic percentages (at%) of Mn, O and C are 0.23%, 10.3% and 89.47%, respectively (Table S1). The high-resolution C 1s and O 1s XPS spectra (Fig. S5b, c) indicate the existence of rich oxygen functional groups and the formation of Mn–O bonds in Mn–O–C [34–36], suggesting that Mn single atoms could be anchored to the graphitic carbon substrate via Mn–O coordination bonds. The high-resolution Mn 2*p* XPS spectrum (Fig. 2a) shows the peaks located at 641.3, 642.8 and 645.5 eV, corresponding to the Mn^2+^, Mn^3+^ and specific shakeup satellite peak [38, 39].

To further determine the valence state of Mn, the X-ray absorption spectroscopy near-edge structure (XANES) spectra were employed. As shown in Fig. 2b, the XANES spectra show the adsorption peak position for Mn–O–C is situated between those for the MnO and MnO_2_, further revealing its typical electronic structure of Mn^δ+^ (2 < *δ* < 4), which is in agreement with the above XPS analysis [38, 39]. The extended X-ray absorption fine structure (EXAFS) curves (Fig. 2c) show the characteristic peak of the Mn–Mn bond at approximately 2.30 Å for the Mn foil and Mn–O bonds at approximately 1.40–1.70 Å for MnO and MnO_2_. The predominant peak at approximately 1.65 Å ascribed to the Mn–O scattering pathway can be observed (Fig. 2c), and there was no peak corresponding to the Mn–Mn scattering pathway, indicating the existence of the single Mn atomic structure. Based on the EXAFS spectra fitting, the coordination numbers of Mn–O are given by CN_Mn-O_ = 4.0 in Mn–O–C sample (Table S2). The simulated EXAFS spectra are well fitted with measured curves (Fig. 2d, e), verifying the rationalization of optimized structure models, confirming the formation of Mn–(O–C_2_)_4_ sites in the Mn–O–C sample (Fig. 2f). Meanwhile, the wavelet transforms (WT) EXAFS oscillation of Mn foil, MnO, MnO_2_ and Mn–O–C was further analyzed to study the atomic configuration. As shown in Fig. 2g-j, the WT contour plots of the Mn–O–C sample display the intensity maximum at approximately 7.0 Å^−1^, corresponding to the Mn–O coordination bond. Moreover, the Mn–Mn signals at approximately 8.0 Å^−1^ are absent as compared to the MnO, MnO_2_ and Mn foil references. The above results clearly prove the formation of precisely regulated Mn–(O–C_2_)_4_ sites, which may endow the Mn–O–C catalyst with high activity and selectivity in catalytic NitRR [34–36].

### NitRR Performance Evaluation

**Fig. 3:**
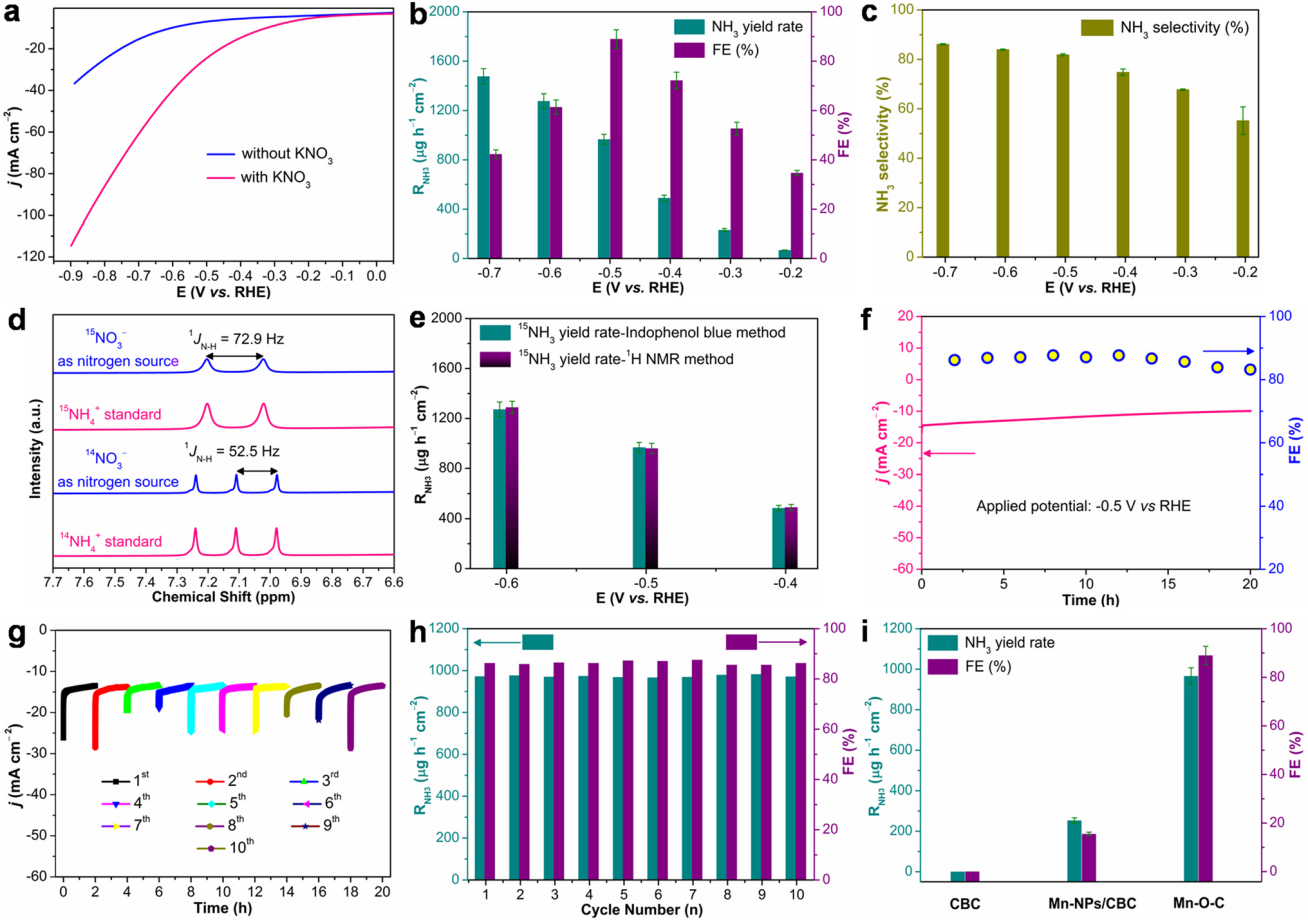
**a** LSV curves of the Mn–O–C in Ar-saturated 0.1 M K_2_SO_4_ and 0.1 M K_2_SO_4_ + 1000 ppm N-KNO_3_ electrolyte. **b** R_NH3_ and FE at each given potential. **c** S_NH3_ of Mn–O–C obtained at different potentials.** d** ^1^H NMR spectra of ^14^NH_4_^+^ and ^15^NH_4_^+^ standards, and the resultant samples from Mn–O–C-catalyzed NitRR using ^14^NO_3_^−^ and ^15^NO_3_^−^ as nitrogen source, respectively. **e** Comparison of R_NH3_ from Mn–O–C using ^15^NO_3_^−^ as nitrogen source at different potentials. **f** Durability test on the applied potential for Mn–O–C. **g** Cycling stability test of Mn–O–C at − 0.5 V (vs. RHE) for 10 cycles with 2.0-h NitRR period per cycle. **h** Corresponding R_NH3_ and FE of each testing cycle. **i** R_NH3_ and FE of CBC, Mn NPs/CBC and Mn–O–C at − 0.5 V (vs. RHE) for 2.0 h NitRR

The NitRR performance of Mn–O–C was evaluated in a typical H-type electrolytic cell under ambient conditions in Ar-saturated 0.1 M K_2_SO_4_ + 1000 ppm N-KNO_3_. We first performed the linear sweep voltammetry (LSV) in Ar-saturated 0.1 M K_2_SO_4_ electrolyte with and without KNO_3_ to study the NitRR catalytic activity of Mn–O–C (Fig. 3a). The obviously enhanced current density under the same potential confirms that nitrate can be effectively reduced by Mn–O–C. It should be noted that before electrocatalytic NitRR by a potentiostatic method, Ar gas was purged to the electrolyte for at least 20 minutes. Figure 3b shows the dependence of NH_3_ yield rate (R_NH3_) and FE on the applied potentials for 2.0 h electrolysis from − 0.2 to − 0.7 V (vs. RHE). The calculated R_NH3_ and FEs based on three repeated experiments are derived from the recorded chronoamperometric curves (Fig. S6a) under different potentials with the yielded NH_3_ being determined by the indophenol blue method (Figs. S6b and S7). The well-established colorimetric methods (Fig. S8) were used to quantify the unreacted NO_3_^−^ in the electrolytes [40, 41]. When applying the more negative potentials from − 0.2 to − 0.7 V (vs. RHE), NH_3_ yield rate can gradually increase. The Mn–O–C electrocatalyst achieves a high R_NH3_ of 1476.9 ± 62.6 μg h^**−**1^ cm^**−**2^ at − 0.7 V (vs. RHE) and a FE of 89.0 ± 3.8% at − 0.5 V (vs. RHE) under ambient conditions, which is comparable to most of recently reported NitRR electrocatalysts (Table S3). Note that the decreased FE here (from − 0.6 V (vs. RHE)) is due to the enhanced competition of the HER at more negative applied potentials [42, 43]. Although the electrodynamic potential of NO_3_^−^ to NO_2_^−^ is higher than that of NO_3_^−^ to NH_3_ [40–43], NO_2_^−^ is an essential intermediate of NitRR and is also quantified by colorimetric method (Fig. S9) [40–43]. As shown in Fig. S10, NO_2_^−^ is almost detectable for the Mn–O–C in all potential ranges, while the FE of NO_2_^−^ is much less than NH_3_, which indicates that NH_3_ is the main product of NitRR. The selectivity of NH_3_ (S_NH3_) of Mn–O–C at different applied potentials was further studied, the R_NH3_ shows the same trend with the increase of potential, and the highest S_NH3_ was 86.1 ± 0.3% (Fig. 3c).

The ^1^H nuclear magnetic resonance (NMR) analysis was subsequently carried out to confirm the yielded NH_3_ exclusively resulted from the Mn–O–C-catalyzed NitRR [40–43]. The experiments were performed using ^14^NO_3_^−^ and ^15^NO_3_^−^ as nitrogen source in Ar-saturated 0.1 M K_2_SO_4_ electrolyte at − 0.5 V (vs. RHE) over a 2.0-h NitRR period. Figure 3d displays the ^1^H NMR spectra of the standards and the yielded ^14^NH_4_^+^ and ^15^NH_4_^+^ in NitRR samples, confirming that the yielded NH_3_ is indeed originated from the Mn–O–C-catalyzed NitRR. The yielded ^15^NH_4_^+^ and concentrations were quantified by both the indophenol blue method (Fig. S7) and ^1^H NMR analysis (Fig. S11). As shown in Figs. 3e and S12a, the yielded ^15^NH_4_^+^ concentrations determined by ^1^H NMR method ranged from − 0.4 to − 0.6 V (vs. RHE), very closely approximated to those determined by the indophenol blue method. Such closely approximated NH_3_ yield rates from ^15^NO_3_^−^ confirmed by the two analytical methods infer that the yielded NH_3_ is indeed originated from the Mn–O–C-catalyzed NitRR.

The control experiments were subsequently conducted to eliminate the environmental interference on the yielded NH_3_ (Fig. S12b). Only ignorable NH_3_ concentration can be detected when the experiments were carried out using Ar-saturated 0.1 M K_2_SO_4_ with ^15^NO_3_^−^ but without applied potential (OCP). The detected NH_3_ concentration is also ignorable using the Mn–O–C catalyst in Ar-saturated 0.1 M K_2_SO_4_ at − 0.5 V (vs. RHE) for 2.0 h (Fig. S12b). The above experimental results eliminate any noticeable environmental interference to the yielded NH_3_ from NitRR. The durability of Mn–O–C for NitRR was evaluated using Ar-saturated 0.1 M K_2_SO_4_ electrolyte at an applied potential of − 0.5 V (vs. RHE) for 20 h (Fig. 3f). It can be seen that the chronoamperometric profile exhibits a slight decrease in the current density during the entire test period and ~ 3.5% loss of FE can be achieved from 2.0 to 20 h, demonstrating high stability of Mn–O–C. The reusability measurement results also indicate that at − 0.5 V (vs. RHE) with a reaction time of 2.0 h for each cycle, no noticeable decay could be observed in the total current density during 10 consecutive cycles (Fig. 3g). Moreover, ignorable change in R_NH3_ and FE (Fig. 3h) further confirms a superior reusability of Mn–O–C. After 10 NitRR measurement cycles, the used Mn–O–C still exhibits atomically dispersed nature of Mn (Figs. S13–S15), no metallic Mn or Mn-related oxides can be detected (Fig. S16), indicating high structural stability of the O–C-coordinated Mn–O–C. In contrast, the catalytic performance of CBC (Fig. S17), Mn NPs/CBC (Fig. S18) and Mn–O–C was further evaluated in Ar-saturated 0.1 M K_2_SO_4_ + 1000 ppm N-KNO_3_ electrolyte. At − 0.5 V (vs. RHE) for 2.0 h of reaction, no NH_3_ product can be detected for CBC, while the Mn NPs/CBC can give a R_NH3_ of 252.8 ± 12.9 μg h^**−**1^ cm^**−**2^ and a FE of 15.5 ± 0.8%, which was much less than Mn–O–C (R_NH3_: 966.1 ± 40.9 μg h^**−**1^ cm^**−**2^ and FE: 89.0 ± 3.8%), implying that the CBC and Mn NPs/CBC are comparatively unfavorable for NitRR (Fig. 3i).

**Fig. 4:**
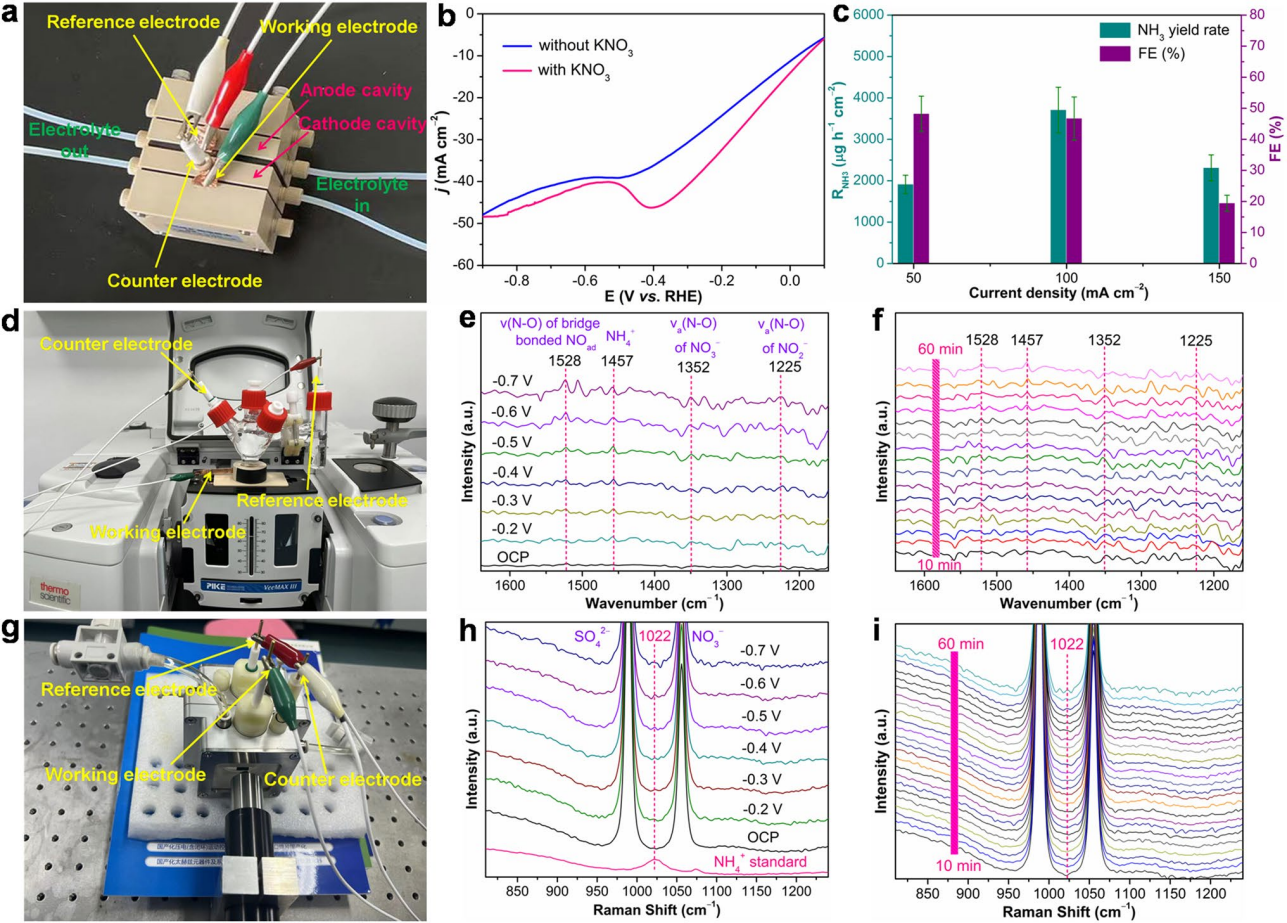
**a** Physical photograph of flow cell reactor for electrocatalytic NitRR. **b** LSV curves of the Mn–O–C in the flow cell with and without NO_3_^**−**^. **c** R_NH3_ and FE at the constant current density of 50, 100 and 150 mA cm^**−**2^. **d** Physical photograph of in situ ATR-SEIRAS reactor for electrocatalytic NitRR.** e** In situ ATR-SEIRAS spectra of electrocatalytic NitRR on Mn–O–C at different potentials. **f** In situ ATR-SEIRAS spectra of Mn–O–C during electrocatalytic NitRR at − 0.5 V (vs. RHE) for 1.0 h. **g** The physical photograph of in situ Raman reactor for electrocatalytic NitRR. **h** In situ Raman spectra of electrocatalytic NitRR on Mn–O–C at different potentials. **i** In situ Raman spectra of Mn–O–C during electrocatalytic NitRR at − 0.5 V (vs. RHE) for 1.0 h

To further evaluate the electrocatalytic NitRR performance, the R_NH3_ of Mn–O–C was determined using a commercial gas diffusion electrode (GDE) (Figs. 4a and S19) [44, 45]. Firstly, LSV of Mn–O–C was conducted in Ar-saturated 0.1 M K_2_SO_4_ solution with and without NO_3_^**−**^, respectively. As shown in Fig. 4b, the current density enhancement with NO_3_^**−**^ over the one without NO_3_^**−**^ indicates that Mn–O–C is active for NitRR catalysis in the flow cell. Moreover, the LSV of Mn–O–C tested in the presence of NO_3_^**−**^ exhibits a remarkable reduction peak at − 0.4 V (vs. RHE) (Fig. 4b), which may be due to the electrochemical reduction of NO_3_^**−**^ [46]. It is worth noting that the significant decrease in current density in the flow cell compared to the H cell (Fig. 3a) may be due to the lack of strong gas (*e.g.*, N_2_, O_2_, CO_2_)–liquid contact significantly enhanced by GDE, and the flow cell can significantly inhibit the competitive HER on the Mn–O–C surface under high current density by continuously cycling the main product ammonia and by-product hydrogen away from the electrode, while also greatly increasing the distance required for nitrate diffusion, lowering the maximum obtainable current densities. The dependence of R_NH3_ and FE at the constant current density of 50, 100 and 150 mA cm^**−**2^ is shown in Fig. 4c. As expected, Mn–O–C shows the highest of R_NH3_ of 3706.7 ± 552.0 μg h^**−**1^ cm^**−**2^ at a current density of 100 mA cm^**−**2^, twofold lager than on the H cell (1476.9 ± 62.6 μg h^**−**1^ cm^**−**2^). As an essential intermediate of NitRR, NO_2_^**−**^ is also found for Mn–O–C at different current densities (Fig. S20). In order to validate the electrocatalytic NitRR mechanism of Mn–O–C, we utilized in situ ATR-SEIRAS to monitor the evolution of NitRR intermediates [47] and the experimental setup and cell are displayed in Figs. 4d and S21. Figure 4e shows the infrared signals when the in situ electrocatalytic NitRR on Mn–O–C during the negative scan from − 0.2 to − 0.7 V (vs. RHE). At the applied potential, the significantly enhanced infrared peaks at 1352 cm^−1^ were assigned to N–O asymmetric stretching vibration of NO_3_^**−**^ [48, 49], which indicates the activation and consumption of NO_3_^**−**^ catalyzed by Mn–O–C. Meanwhile, the infrared peaks at 1225 cm^−1^ were attributed to N–O antisymmetric stretching vibration of NO_2_^**−**^ [48, 50], confirming the formation of by-product NO_2_^**−**^ from NitRR, which is also consistent with the electrochemical experimental results. Interestingly, the vibration bands of adsorbed NO in bridge adsorption mode were detected at 1528 cm^−1^ [48, 51]. In addition, the gradually enhanced infrared peaks at 1457 cm^−1^, which can be ascribed to N–H bending vibration of NH_4_^+^ [50, 52]. Based on the in situ ATR-SEIRAS analysis, we proposed the following pathway for the NitRR on Mn–O–C surface: NO_3_^−^  → *NO_3_ → *NO_2_ → *NO → *NH_2_OH → *NH_3_. We further conducted in situ ATR-SEIRAS measurement at − 0.5 V (vs. RHE) for 1.0-h NitRR. The intensity of the characteristic peaks at 1225, 1352, 1457 and 1528 cm^−1^ is increased obviously from 10 to 60 min, implying that the NitRR takes place gradually with reaction time under the given electrocatalytic conditions (Figs. 4f and S22). We also performed in situ Raman spectroscopy tests in Ar-saturated 0.1 M K_2_SO_4_ solution with NO_3_^**−**^ electrolyte (Fig. 4g). As shown in Fig. 4h, when OCP was applied, two main Raman peaks at 989 and 1056 cm^−1^ were attributed to SO_4_^2−^ and NO_3_^−^, respectively. As the applied potential is scanned from OCP to − 0.7 V (vs. RHE), a new signal appears at 1022 cm^−1^ which is in accordance with NH_4_^+^ standard, confirming the formation of NH_3_ from NitRR. We further investigated the electrochemical NitRR at − 0.5 V (vs. RHE) for 1.0 h by the in situ Raman spectroscopy measurements (Fig. 4i)*.* It was also found that the Raman intensity of the peaks belonging to NH_4_^+^ at around 1022 cm^−1^ gradually enhanced from 10 to 60 min. The above in situ ATR-SEIRAS and Raman spectroscopy measurements confirm the successful realization of electrocatalytic NitRR to NH_3_ over Mn–O–C in this work.

### DFT Prediction of NitRR Activity of Mn–(O–C_2_)_4_ Site

**Fig. 5:**
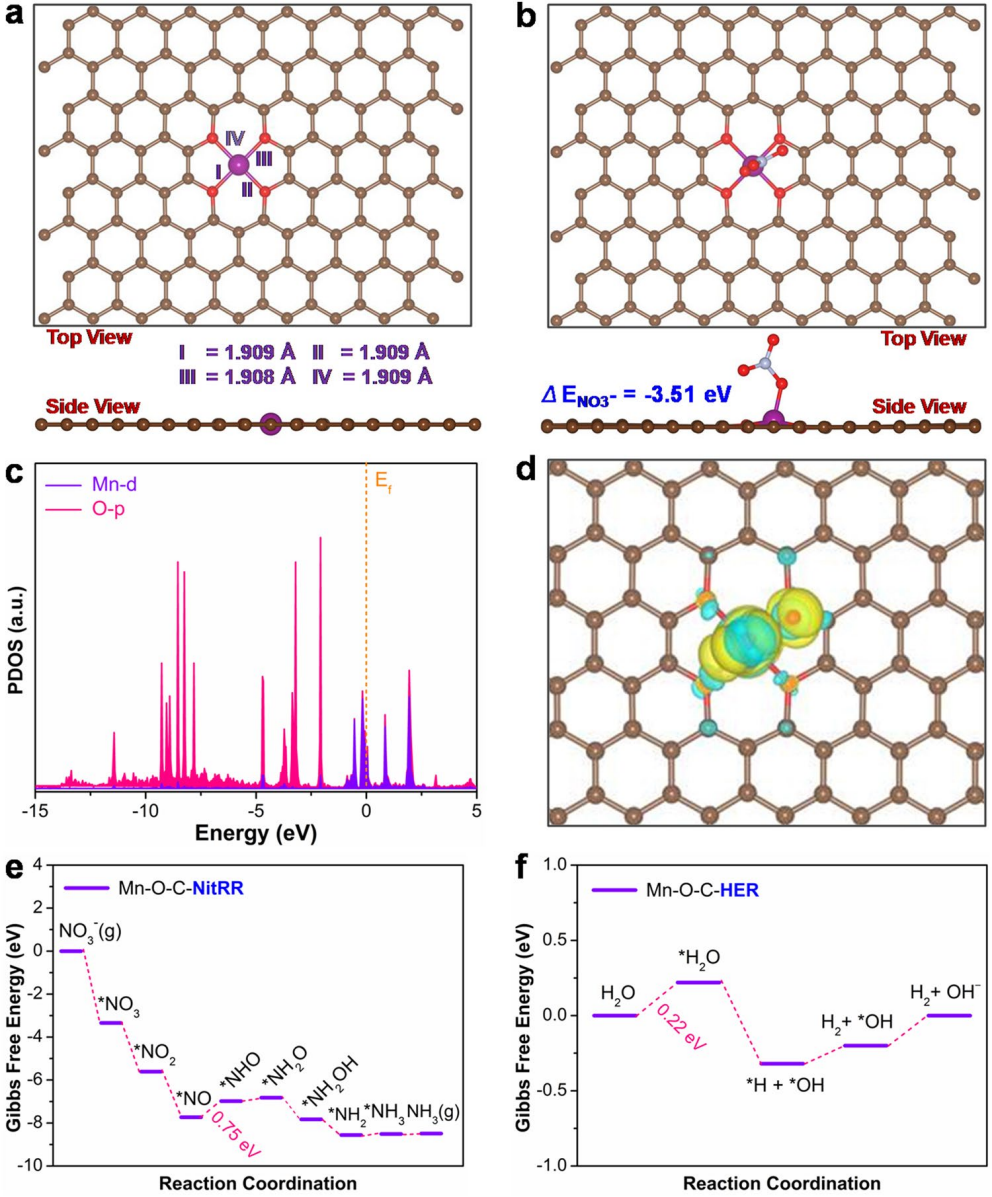
**a** DFT optimized configurations of Mn–(O–C_2_)_4_. **b** NO_3_^−^ adsorption on Mn–(O–C_2_)_4_. **c** The computed projected density of states (PDOS) of Mn–(O–C_2_)_4_ after NO_3_^−^ adsorption. The Fermi level was set to be as denoted by the orange dashed line. **d** The optimized charge density difference of the Mn–(O–C_2_)_4_ after adsorbing NO_3_^−^.** e**,** f** Gibbs free energy diagram of NitRR to NH_3_ and water dissociation on Mn–(O–C_2_)_4_

Based on the aforementioned in situ ATR-SEIRAS results, we performed DFT calculations to understand the electrochemical NitRR mechanism on Mn–O–C catalyst. Figure 5a shows the DFT optimized Mn–(O–C_2_)_4_ configuration on graphitic carbon with four Mn–O bond lengths of 1.909, 1.909, 1.908 and 1.909 Å. It is known that adequate NO_3_^−^ adsorption and activation on catalyst are the essential prerequisite for an efficient NitRR process. Thus, NO_3_^−^ adsorption on the Mn–(O–C_2_)_4_ unit was evaluated via theoretical adsorption energy. As shown in Fig. 5b, the adsorption energy of NO_3_^−^ adsorbed on Mn–(O–C_2_)_4_-coverage graphene is − 3.51 eV, which indicates the strong interaction between NO_3_^−^ and the Mn-SAs/CBC catalyst. Moreover, the projected density of states (PDOS) of the NO_3_^−^ bonded to the catalytic sites (Fig. 5c) exhibit a significant hybridization near the Fermi level, and the O 2p orbital shows a vast majority overlap with the Mn 3d orbital according to the charge density difference calculation (Fig. 5d). The result confirms the role of Mn in NitRR adsorption and further activation via electronic donation [31, 53]. The Gibbs free energies diagram further provides more details about the NitRR mechanism on the Mn–(O–C_2_)_4_ site. As shown in Figs. 5e and S23, NO_3_^−^ is first adsorbed to give *NO_3_ with a dramatic energy decrease up to − 3.34 eV, implying favorable NO_3_^−^ adsorption. *NO_3_ spontaneously decomposed into *NO_2_ with an energy release of − 2.27 eV. Then, by adsorbing a proton to couple with an electron transfer, one N–O bond in *NO_2_ would be broken, and *NO_2_ transformed to *NO with a downhill free energy change of − 2.12 eV. Next, three continued protonation steps of *NO generate *NHO, *NH_2_O and *NH_2_OH, respectively. The rate-determining step over Mn–(O–C_2_)_4_ was the formation of *NO to *NHO (*NO → *NHO) with a free energy increase of 0.75 eV, which was much higher than the energy increase of *NHO to *NH_2_O (0.17 eV). Subsequently, *NH_2_OH goes through another N–O cleavage and hydrogenation to form *NH_3_. Finally, *NH_3_ desorbs form the Mn–O–C catalyst surface to produce free NH_3_ via consuming energy of 0.02 eV. Besides, the Gibbs free energies of the HER process on the Mn–(O–C_2_)_4_ site were further calculated (Figs. 5f and S24). However, the free energy of H_2_O adsorbed to form *H_2_O is uphill and the value is 0.22 eV, which is much higher than NO_3_^−^ adsorption to form *NO_3_ (**− **3.34 eV). Therefore, the isolated Mn–(O–C_2_)_4_ sites are beneficial for active adsorption of NO_3_^−^, the favorable formation of *NHO, and suppression of the competition from the HER.

## Conclusions

In this work, an atomically dispersed and oxygen-coordinated Mn–O–C catalyst has been developed through an impregnation–pyrolysis–etching synthetic approach, which is effective to significantly regulate the density active sites. The high activity of the Mn–O–C catalyst was evidenced by a superior R_NH3_ of 1476.9 ± 62.6 μg h^**−**1^ cm^**−**2^ at − 0.7 V (vs. RHE) and a FE of 89.0 ± 3.8% at − 0.5 V (vs. RHE) under ambient conditions. An in situ FT-IR and Raman spectroscopic investigations combined with DFT calculations found that the exclusive existence of Mn–(O–C_2_)_4_ sites can effectively suppress the competitive HER and greatly promote the adsorption of reacting intermediates and thus high NitRR performance. The current research results also show that nitrogen pollution management is expected to be realized through the electrocatalytic approach of using single-site Mn catalysts, thus contributing to the future sustainability of fertilizer and renewable fuel recovery in many aspects.

## Supplementary Information

Below is the link to the electronic supplementary material.

Supplementary file1 (PDF 2279 kb)
