# Supplementary material for: Oxygen-Coordinated Single Mn Sites for Efficient Electrocatalytic Nitrate Reduction to Ammonia
Source: Nanomicro Lett. 2023 Nov 6;16:9. doi: 10.1007/s40820-023-01217-z (PMC10628069; doi:10.1007/s40820-023-01217-z)
Supplement: Supplementary file 1 — Supplementary file1 (PDF 2279 kb) [file 40820_2023_1217_MOESM1_ESM.pdf]

Supporting Information for

Oxygen-Coordinated Single Mn Sites for Efficient Electrocatalytic Nitrate Reduction to Ammonia

Shengbo Zhang[1, 2], Yuankang Zha[1, 2], Yixing Ye[1, 2], Ke Li[3*], Yue Lin[4], Lirong Zheng[5], Guozhong Wang[1, 2], Yunxia Zhang[1, 2], Huajie Yin[1, 2], Tongfei Shi[1, 2, *], Haimin Zhang[1, 2, *]

[1]Laboratory of Materials Physics, Centre for Environmental and Energy Nanomaterials, Anhui Key Laboratory of Nanomaterials and Nanotechnology, CAS Center for Excellence in Nanoscience, Institute of Solid State Physics, HFIPS, Chinese Academy of Sciences, Hefei 230031, P. R. China

[2]University of Science and Technology of China, Hefei 230026, P. R. China

[3]Key Laboratory of Agricultural Sensors, Ministry of Agriculture, School of Information and Computer, Anhui Agricultural University, Hefei 230026, P. R. China

[4]Hefei National Research Center for Physical Sciences at the Microscale, University of Science and Technology of China, Hefei 230026, P. R. China

[5]Beijing Synchrotron Radiation Facility, Institute of High Energy Physics, Chinese Academy of Sciences, 19B Yuquan Road, Beijing 100049, P. R. China

*Corresponding authors. E-mail: zhanghm@issp.ac.cn (Haimin Zhang); keli@ahau. edu.cn (Ke Li); tfshi@issp.ac.cn (Tongfei Shi)

S1 Experimental Section

S1.1 Materials

Bacterial cellulose (BC) pellicle was obtained from Guilin Qihong Technology Co., Ltd., China. $MnSO_4·4H_2O$ were purchased from Sinopharm Chemical Reagent Co., Ltd. KSO_4 (99.0%), KNO_3 (99.0%), sodium nitroferricyanide(III) dehydrate ($C_5FeN_6Na_2O·2H_2O$, 99.0%), sodium citrate ($C_6H_5Na_3O_7·2H_2O$, 99.0%), NaOH (96.0%), salicylic acid ($C_7H_6O_3$, 99.5%), NaClO (available chlorine ≥ 5.0%), NH_4Cl (99.5%), thiosemicarbazide (CH_5N_3S, 99.0%), *p*-aminobenzenesuifonamide ($NH_2C_6H_4SO_2NH_2$, 95.0%), N-(1-naphthyl) ethylenediamine dihydrochloride ($C_{10}H_7NHC_2H_4NH_2·2HCl$, 95.0%), $^{15}KNO_3$ (AR), $^{14}NH_4Cl$ (AR), $^{15}NH_4Cl$ (AR), were purchased from Shanghai Aladdin Biochemical Technology Co., Ltd. Shanghai. All solutions were prepared using deionized water (Millipore Corp., 18.2 MΩ cm). Commercial carbon paper (CP, HCP030N) was purchased from Shanghai Hesen Electric Co. Ltd.

S1.2 Material Characterization

X-ray diffraction (XRD) patterns were acquired using Philips X'pert PRO with Cu Ka radiation (λ = 1.5418 Å) at 40 kV and 40 mA. FT-IR measurements were conducted by a Nicolet Nexus FT-IR spectrometer. Raman spectra were recorded by a Renishaw Micro-Raman Spectroscopy (Renishaw in Via Reflex) with 532 nm excitation laser. The scanning electron microscopy (SEM) images were obtained using SU8020 (Hitachi, Japan). The transmission electron microscopy (TEM) images were obtained using JEMARM 200F. The aberration-corrected high-angle annular dark-field scanning transmission electron microscopy (HAADF-STEM) measurements and EDX spectroscopy were performed on a JEM-ARM200F. The X-ray photoelectron spectroscopy (XPS) spectra were obtained using an ESCALAB 250 X-ray photoelectron spectrometer (Thermo, America). Nitrogen adsorption-desorption isotherms

were measured using Autosorb-iQ-Cx. The synchrotron-based X-ray X-ray absorption near-edge structure (XANES) and the extended X-ray absorption fine structure (EXAFS) measurements were performed at the 1W1B station of Beijing Synchrotron Radiation Facility, China. The EXAFS data were processed according to the standard procedures using the ATHENA module implemented in the IFEFFIT software packages. Mn^{2+} and metallic Mn contents were quantitatively determined by ICP-AES (ICP-6300, Thermo Fisher Scientific).

S1.3 Calculation of NitRR Performance

S_{NH_3}, R_{NH_3} and FE are calculated by the following equations:

$$S_{NH_3}\,(\%) = \frac{\Delta C_{N\text{-}NH_3}}{\Delta C_{N\text{-}NO_3^-}} \times 100\% \tag{S1}$$

$$R_{NH_3}\,(\mu g\ h^{-1}\ mg_{cat.}^{-1}) = \frac{C_{NH_3}(\mu g\ mL^{-1}) \times V(mL)}{t\ (h) \times m_{cat.}\ (mg)} \tag{S2}$$

$$FE\ (\%) = \frac{8 \times n_{NH_3}(mol) \times F\ (C\ mol^{-1})}{Q\ (C)} \times 100\% \tag{S3}$$

where, $\Delta C_{N\text{-}NH_3}$ is the generated concentration of N-NH_3, $\Delta C_{N\text{-}NO_3^-}$ is the difference in N-NO_3^- concentration before and after electrolysis. C_{NH_3} and V are the measured NH_3 concentration and the electrolyte solution volume, respectively. t is the electrolysis period and $m_{cat.}$ is the amount of the loaded electrocatalyst, F is the faradaic constant (96485 C mol^{-1}) and Q is the total charge transferred during electrolysis period.

$R_{NO_2^-}$ and FE are calculated by the following equations:

$$R_{NO_2^-}\,(\mu g\ h^{-1}\ mg_{cat.}^{-1}) = \frac{C_{NO_2^-}(\mu g\ mL^{-1}) \times V(mL)}{t\ (h) \times m_{cat.}\ (mg)} \tag{S4}$$

$$FE\ (\%) = \frac{2 \times n_{NO_2^-}(mol) \times F\ (C\ mol^{-1})}{Q\ (C)} \times 100\% \tag{S5}$$

where, $C_{NO_2^-}$ and V are the measured NO_2^- concentration and the electrolyte solution volume, respectively, t is the electrolysis period and $m_{cat.}$ is the amount of the loaded electrocatalyst, F is the faradaic constant (96485 C mol^{-1}) and Q is the total charge transferred during electrolysis period.

S1.4 ^{15}N Isotope Labelling Experiments

For quality assurance required, ^{15}N isotopic labelling experiments were conducted using 0.1 M K_2SO_4 + 1000 ppm N-$K^{15}NO_3$ as the electrolyte with identical experimental procedure as that of Ar-saturated 0.1 M K_2SO_4 + 1000 ppm N-KNO_3 experiments. For ^1H NMR method, D_2O (99.9 atom% D, Aladdin Biochemical Technology Co., Ltd. Shanghai) used as internal standard. The yielded $^{15}NH_3$ and $^{14}NH_3$ were analyzed by the ^1H NMR methods using Bruker Avance-400 MHZ.

S1.5 DFT Calculations

The first-principle calculations were performed within the framework of DFT as implemented in the Vienna Ab initio Simulation Package (VASP) [S1]. The projector augmented wave (PAW) method has been used to describe the inert core electrons [S2]. A cut off energy of 500 eV was used for the expansion of the wave functions. The electronic exchange-correlation effects were described with Perdew-Burke-Ernzerhof generalized gradient approximation (PBE-GGA) functional [S3, S4]. A vacuum of 15 Å in the z-direction is used to avoid strong

interaction between the adsorbate and periodic images. The k-point sampling was cantered 3 × 3 × 1 mesh form the Monkhorst-Pack scheme. All relaxations were done until the force of the system converges to 0.02 eV/Å. The free energy of a gas phase molecule and the adsorbates on the constructed model are calculated according to the formula: $G = E + \text{ZPE} - TS$, where E is the total energy, ZPE is the zero-point energy, T is the temperature in kelvin (298.15 K used here), and S is the entropy.

S2 Supplementary Figures and Tables

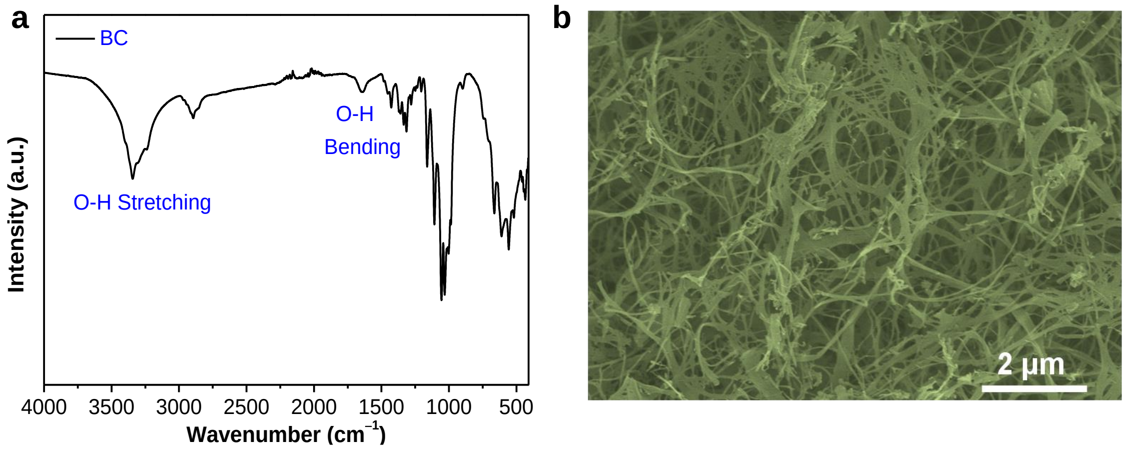

Fig. S1 (**a**) FT-IR spectrum and (**b**) SEM image of pre-treated BC

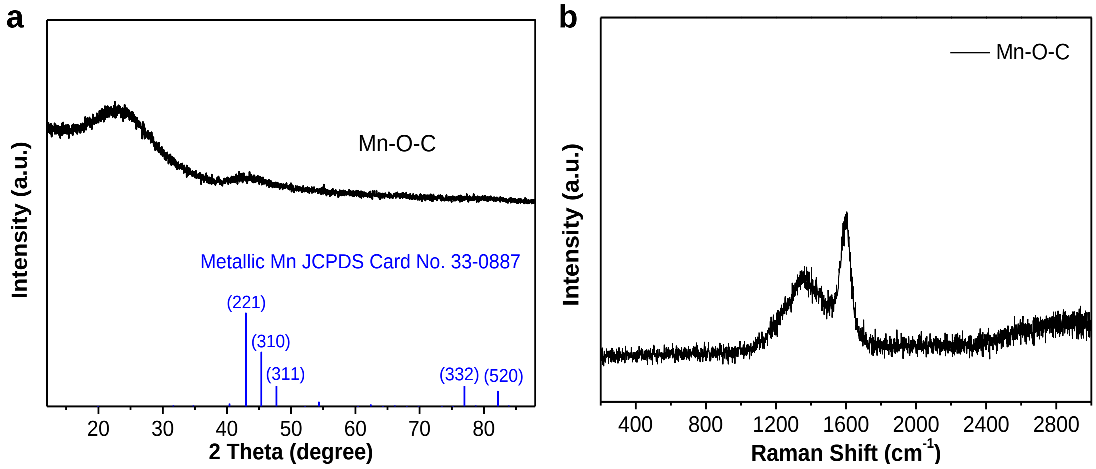

Fig. S2 (**a**) XRD pattern and (**b**) Raman spectrum of Mn-O-C

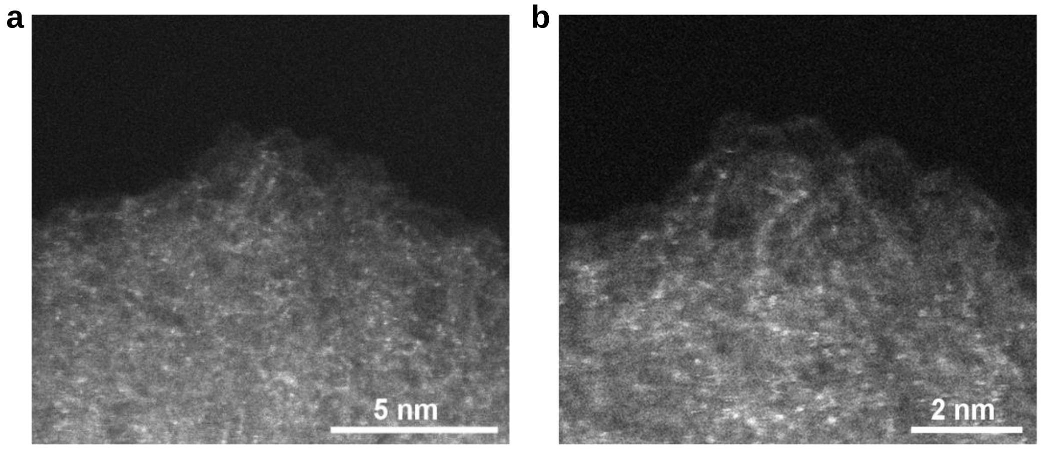

Fig. S3 Aberration-corrected HAADF-STEM images obtained from different locations of Mn-O-C

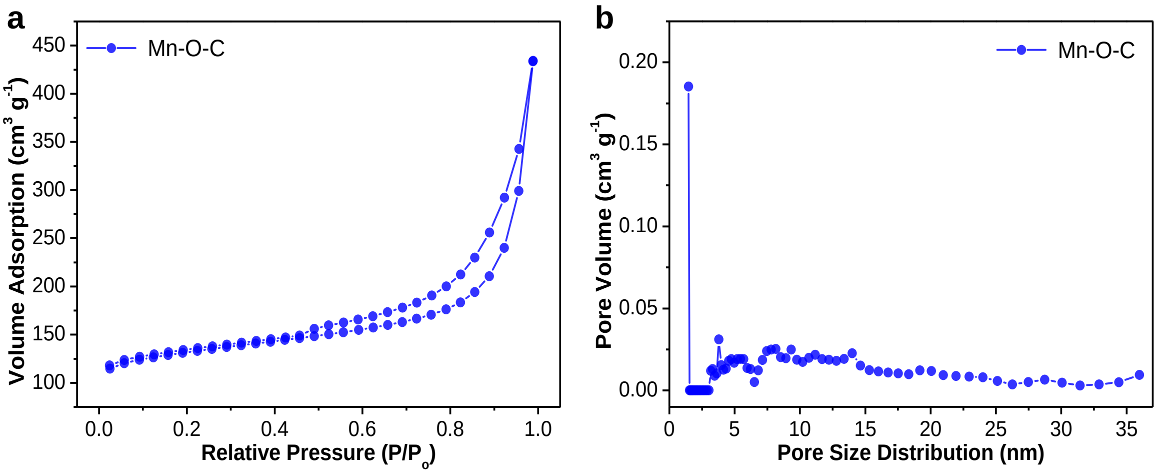

Fig. S4 (**a**) N_2 adsorption-desorption isotherm and (**b**) corresponding pore size distribution curve of Mn-O-C

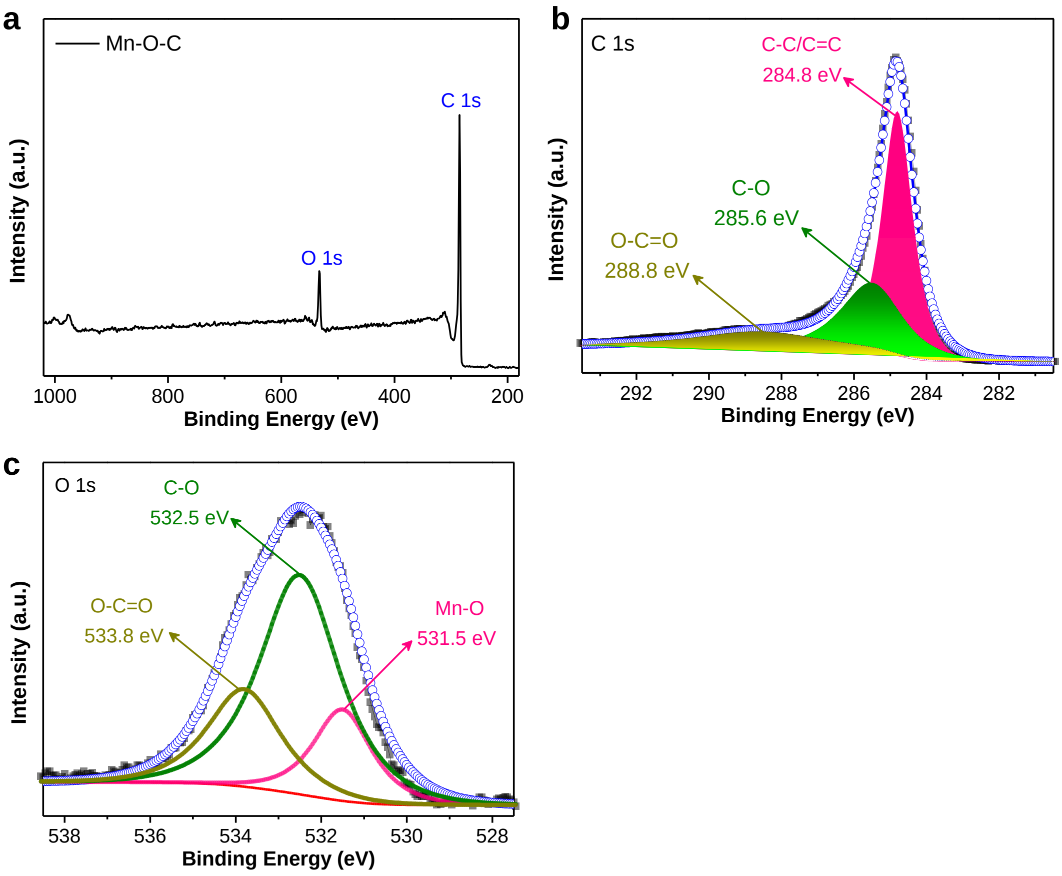

Fig. S5 (**a**) Survey XPS spectrum and high-resolution XPS spectra of (**b**) C 1*s* and (**c**) O 1*s* of Mn-O-C

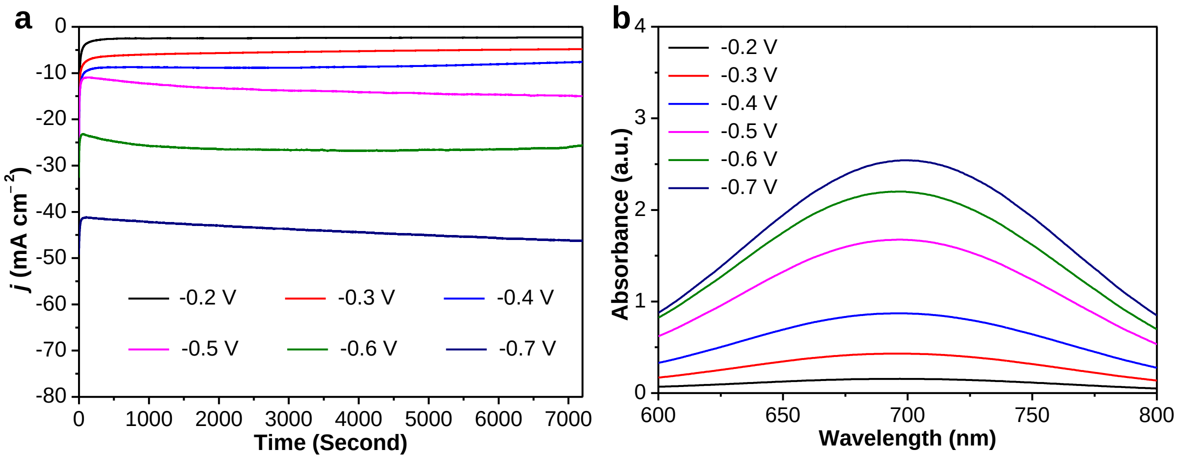

Fig. S6 (**a**) Time-dependent current density curves at different potentials in 0.1 M K_2SO_4 + 1000 ppm N-KNO_3 electrolyte over a 2.0 h period. (**b**) UV-Vis absorption spectra of the corresponding samples

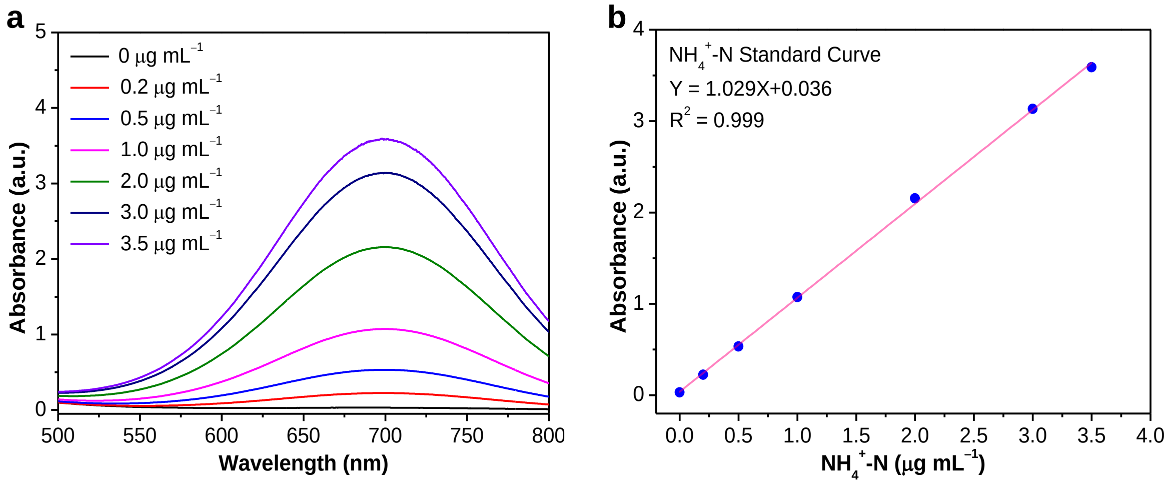

Fig. S7 (**a**) UV-Vis absorption spectra obtained from the solutions with different NH_4^+-N concentrations (0, 0.2, 0.5, 1.0, 2.0, 3.0 and 3.5 μg mL^{-1}). (**b**) Calibration curve used to determine NH_4^+-N concentration

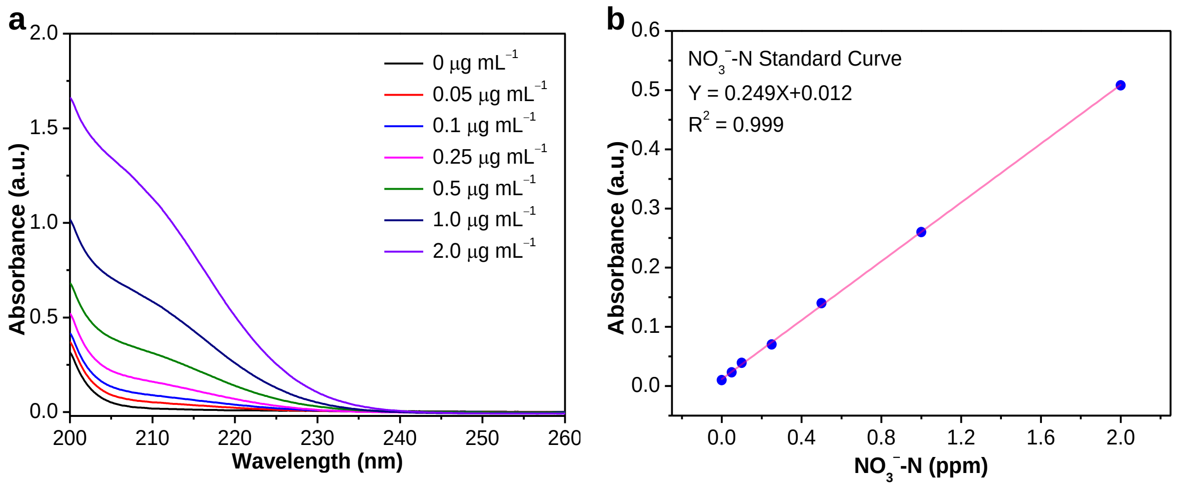

Fig. S8 (**a**) UV-Vis absorption spectra obtained from the solutions with different NO_3^--N concentrations (0, 0.05, 0.1, 0.25, 0.5, 1.0 and 2.0 μg mL^{-1}). (**b**) Calibration curve used to determine NO_3^--N concentration

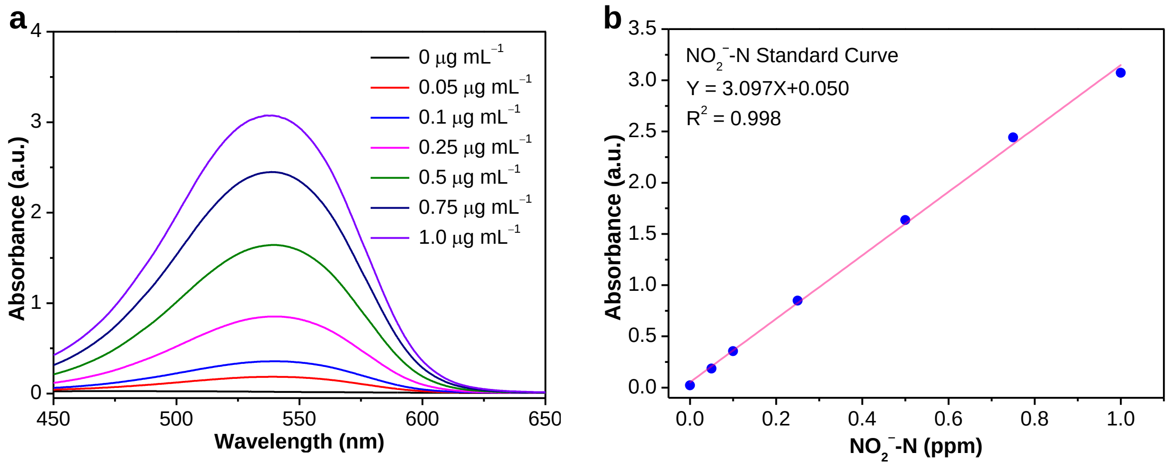

Fig. S9 (**a**) UV-Vis absorption spectra obtained from the solutions with different NO_2^--N concentrations (0, 0.05, 0.1, 0.25, 0.5, 0.75 and 1.0 μg mL^{-1}). (**b**) Calibration curve used to determine NO_2^--N concentration

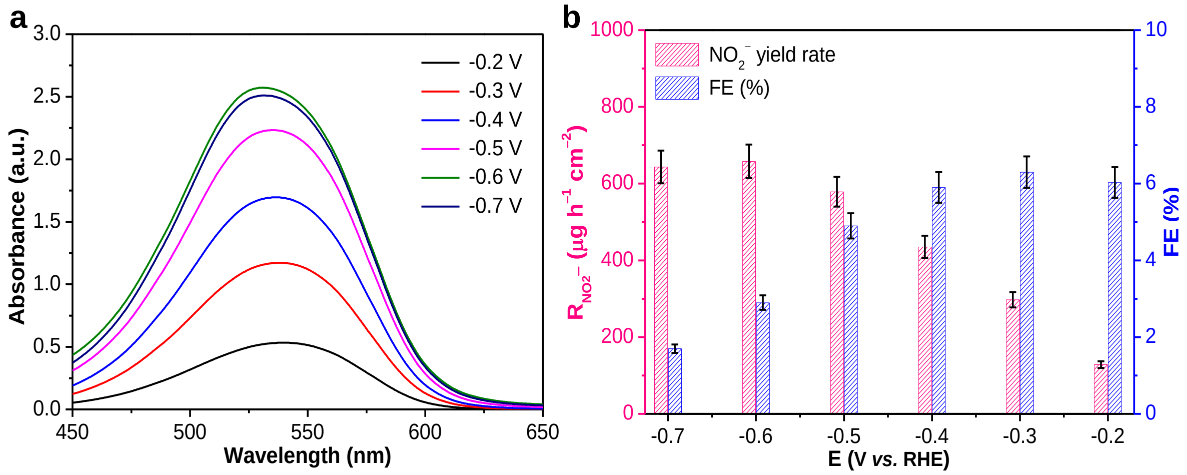

Fig. S10 (**a**) UV-Vis absorption spectra obtained at different potentials in Ar-saturated 0.1 M K_2SO_4 + 1000 ppm N-KNO_3 electrolyte over a 2.0 h period. (**b**) $R_{NO_2^-}$ and FE at each given potential

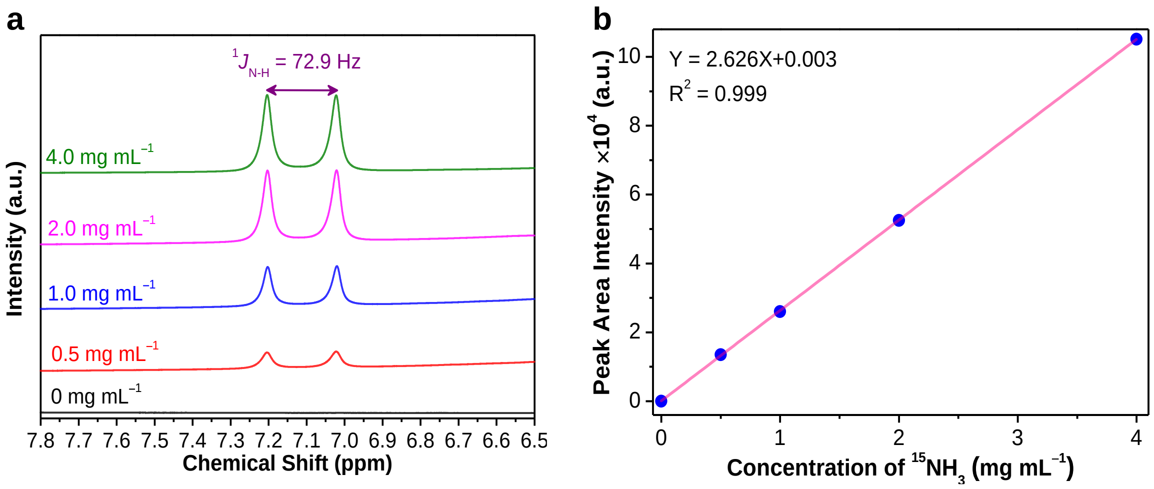

Fig. S11 (**a**) ^{1}H NMR spectra of $^{15}NH_3$ standards. (**b**) The corresponding $^{15}NH_3$ calibration curve

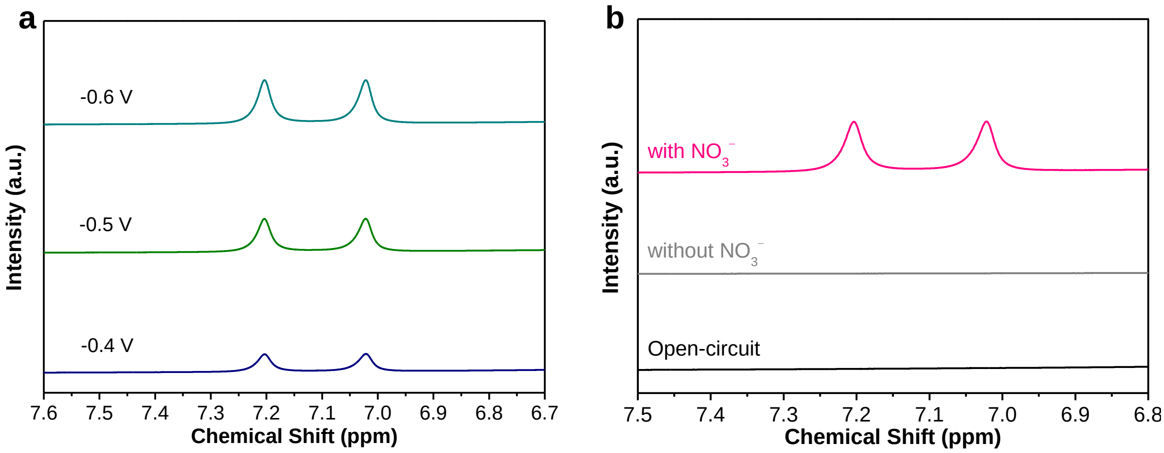

Fig. S12 (**a**) ^{1}H NMR spectra of the yielded $^{15}NH_4^+$ by Mn-O-C in Ar-saturated 0.1 M K_2SO_4 electrolyte for 2.0 h period with $^{15}NO_3^-$ as nitrogen source at different potentials of −0.4 V, −0.5 V and −0.6 V (*vs.* RHE). (**b**) ^{1}H NMR spectra of the collected samples after 2.0 h NtRR in Ar-saturated 0.1 M K_2SO_4 solution at −0.5 V (*vs.* RHE) with and without $^{15}NO_3^-$, Ar-saturated 0.1 M K_2SO_4 + $^{15}NO_3^-$ solution under open-circuit potential (OCP)

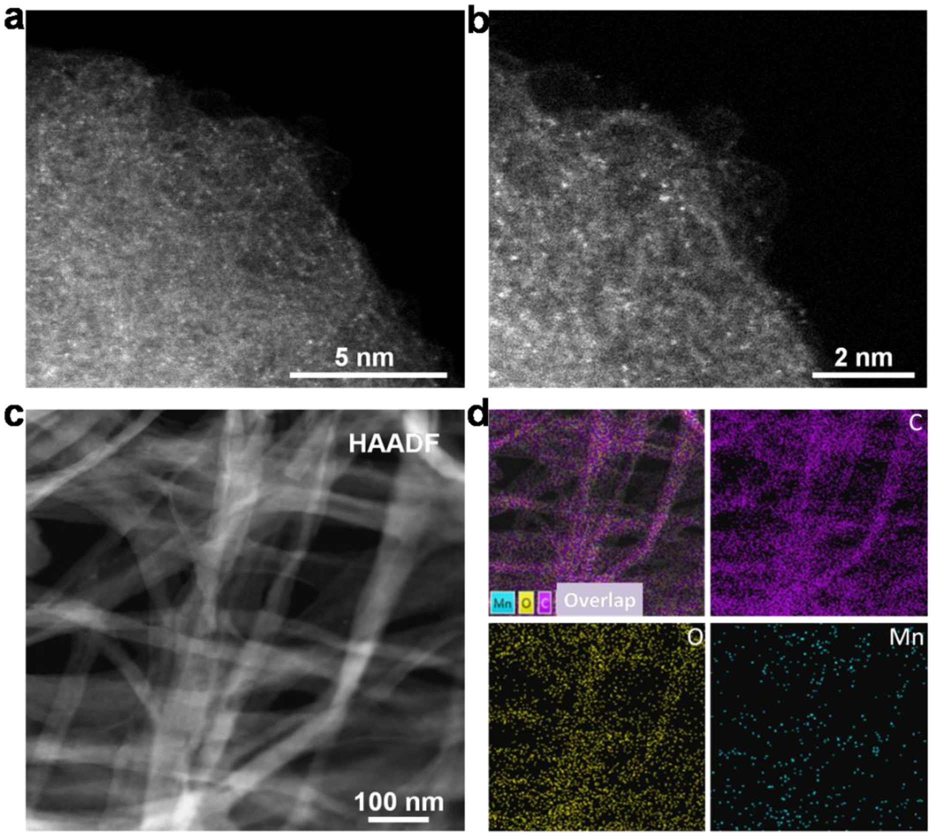

Fig. S13 (**a**) and (**b**) Enlarged aberration-corrected HAADF-STEM images of Mn-O-C after 10 NitRR recycles. (**c**) The HAADF-STEM image and (**d**) corresponding elemental mapping images of Mn-O-C after 10 NitRR recycles

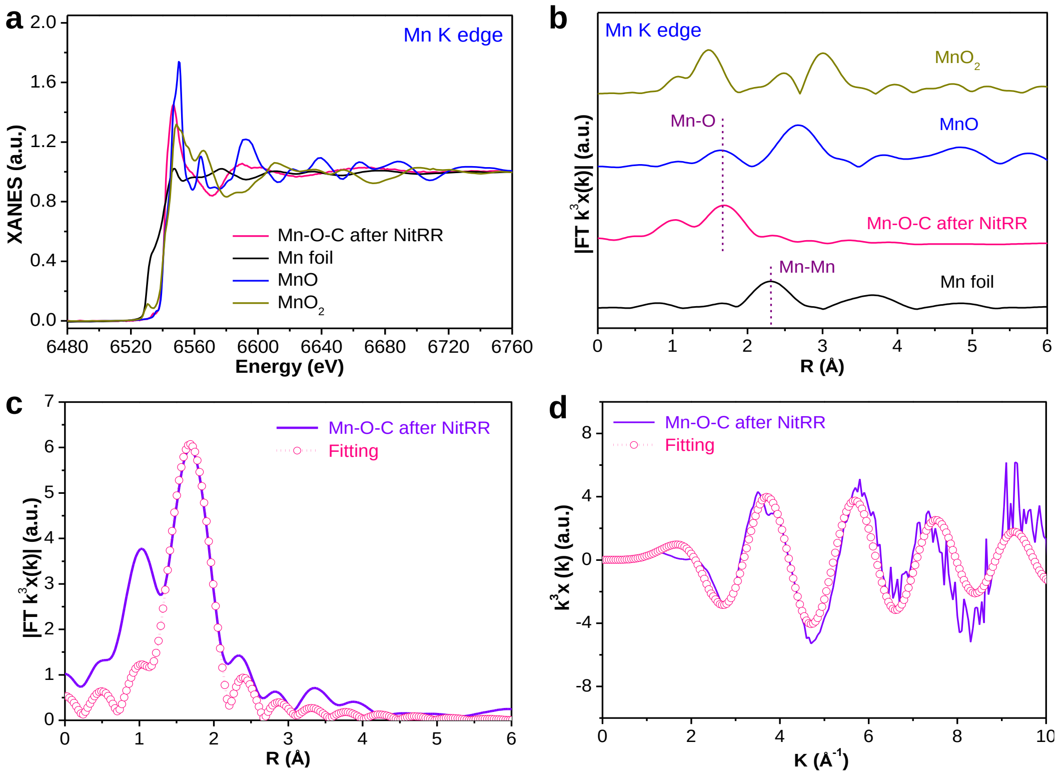

Fig. S14 (**a**) Mn *K* edge XANES spectra and (**b**) k^3-weighted FT-EXAFS spectra of Mn-O-C after 10 NitRR recycles and references. Mn *K* edge EXAFS fitting curves of Mn-O-C after 10 NitRR recycles at (**c**) *R* space and (**d**) *k* space

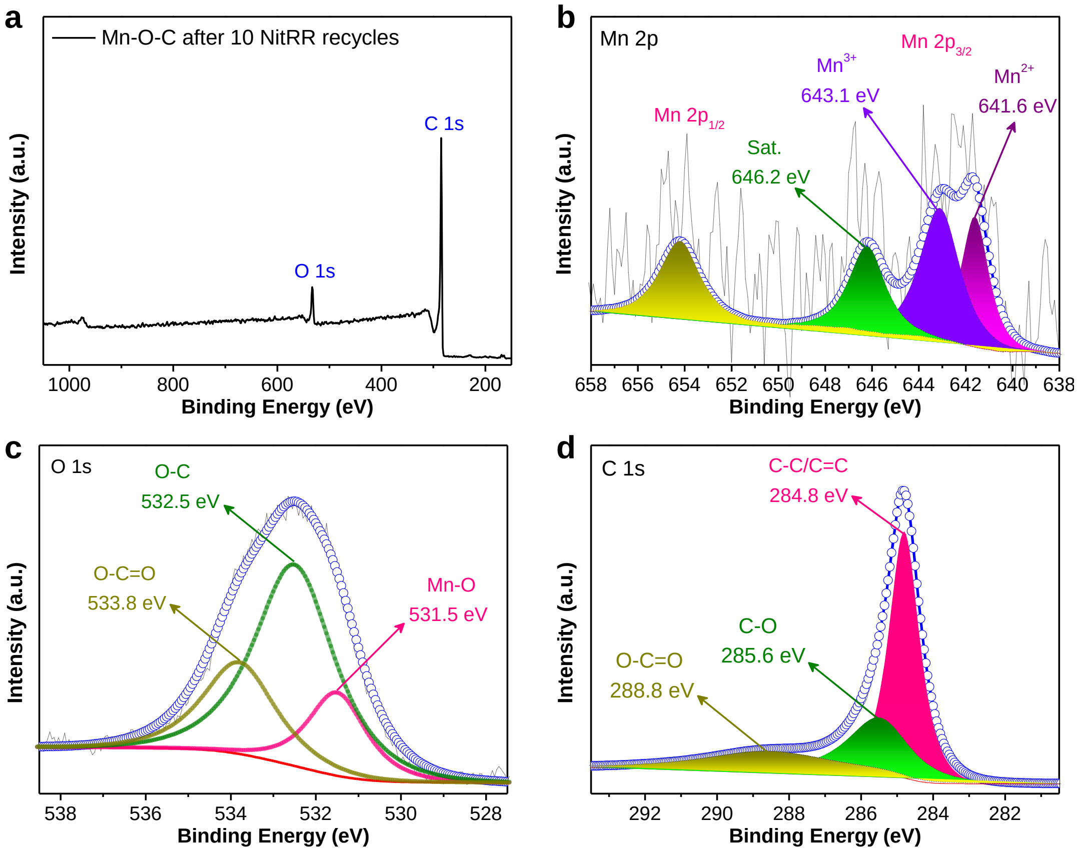

Fig. S15 (**a**) Survey XPS spectrum and high-resolution XPS spectra of (**b**) Mn 2*p*, (**c**) O 1*s*, (**d**) C 1*s* of Mn-O-C after 10 NitRR recycles

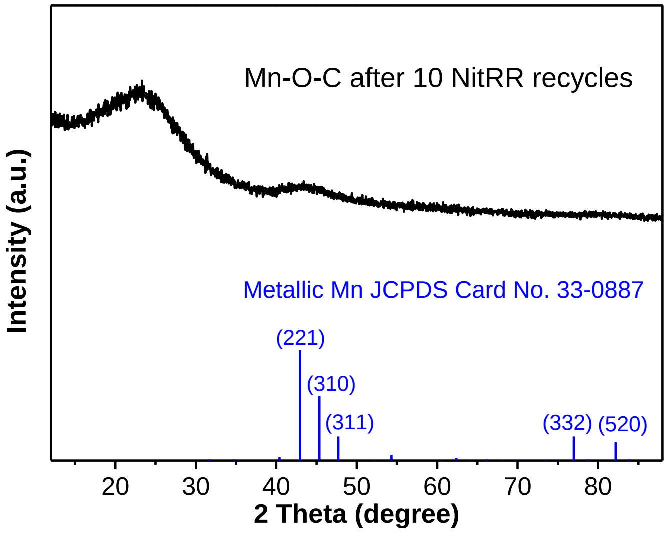

Fig. S16 XRD patterns of Mn-O-C after 10 NitRR recycles

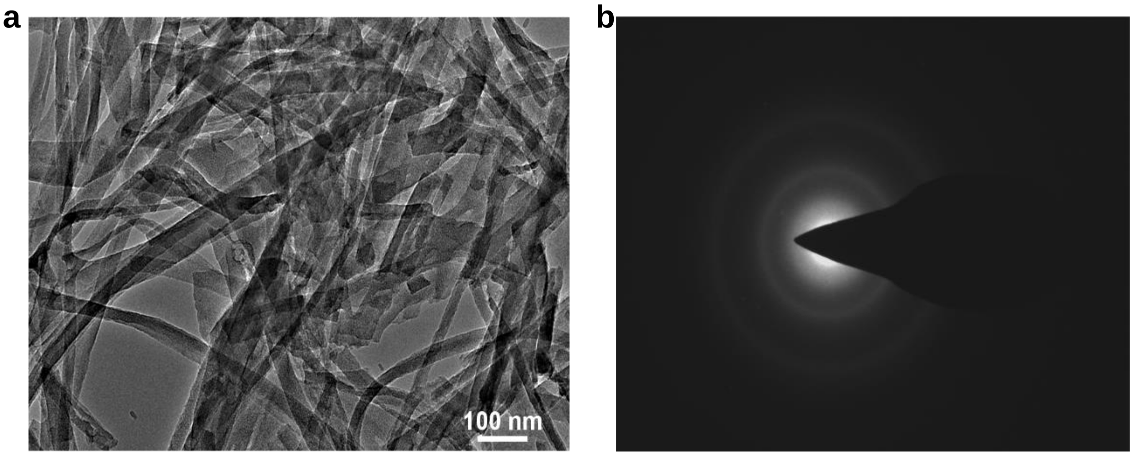

Fig. S17 (**a**) TEM image and (**b**) SAED pattern of CBC

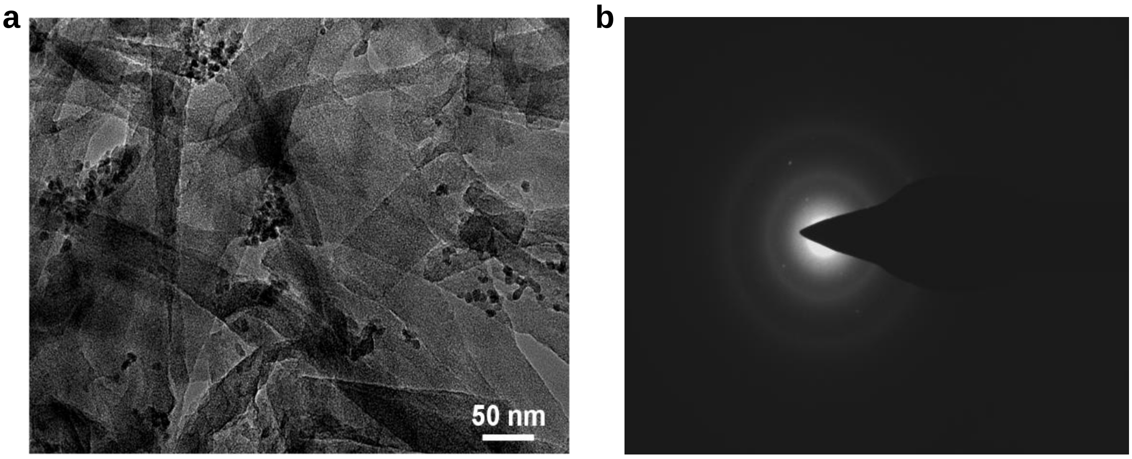

Fig. S18 (**a**) TEM image and (**b**) SAED pattern of Mn-NPs/CBC

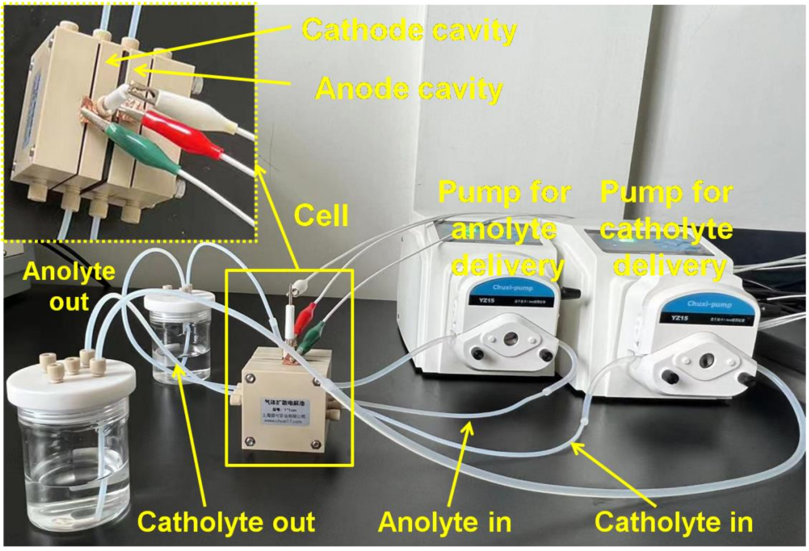

Fig. S19 The physical photographs of flow-cell reaction system and reactor for electrocatalytic NitRR

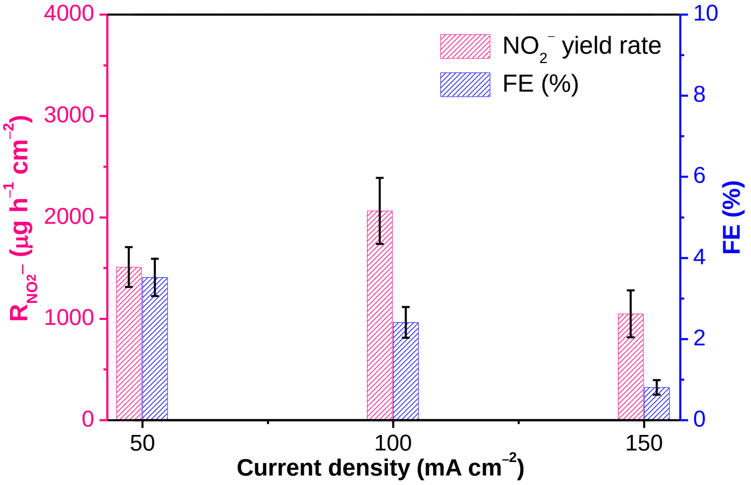

Fig. S20 $R_{NO_2^-}$ and FE at the constant current density of 50, 100 and 150 mA cm^{-2}

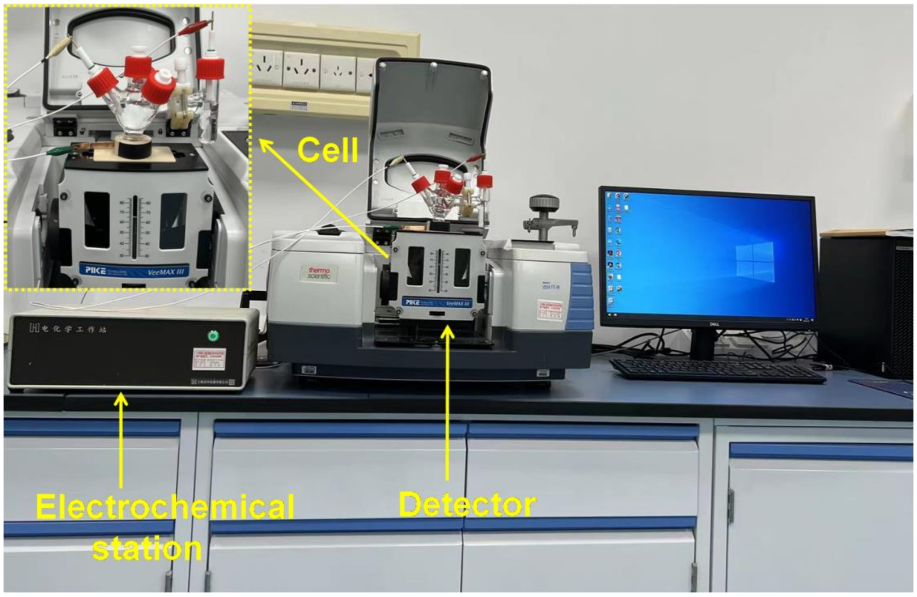

Fig. S21 The physical photographs of *in-situ* ATR-SEIRAS measurements system and reactor for electrocatalytic NitRR

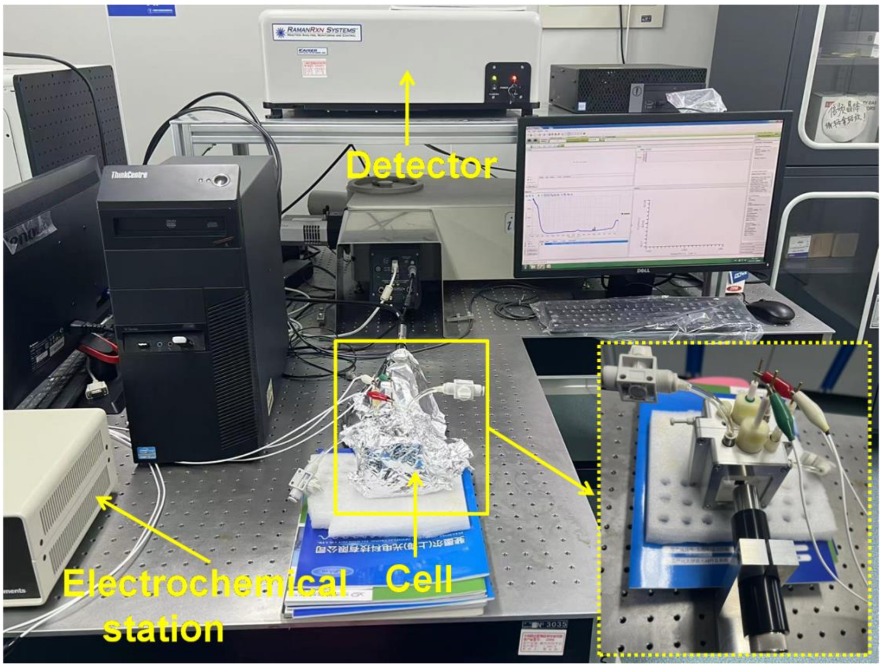

Fig. S22 The physical photographs of *in-situ* Raman measurements system and reactor for electrocatalytic NitRR

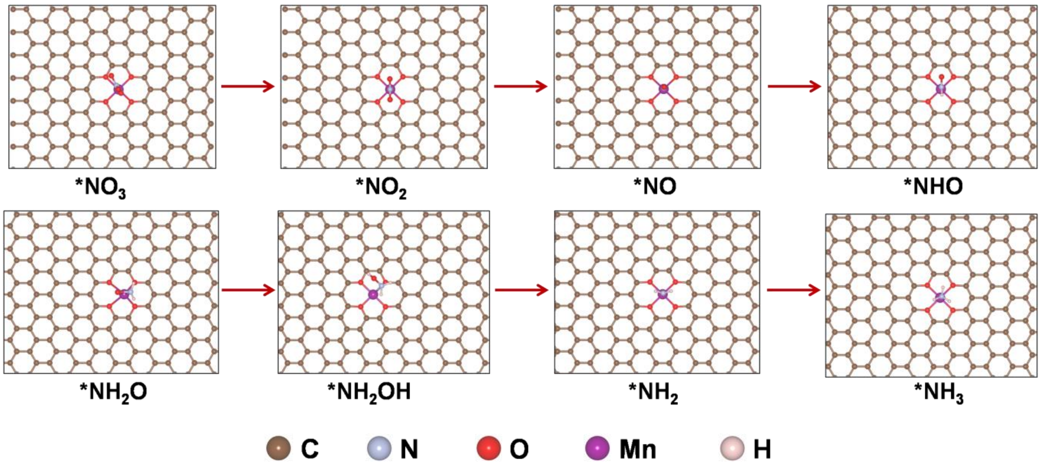

Fig. S23 Top view of corresponding intermediates structures for each step

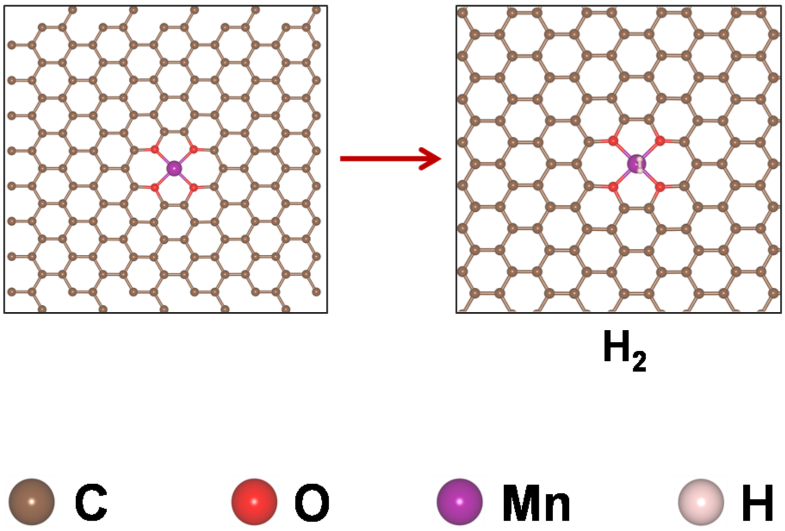

Fig. S24 Top view of corresponding intermediates structures for each step

Table S1 The atomic percentage of each element in the as-prepared Mn-O-C sample obtained by XPS

Elements	Mn-O-C
Mn (at%)	0.23
O (at%)	10.3
C (at%)	89.47

Table S2 Structural parameters extracted from the Mn *K*-edge EXAFS fitting data of Mn-O-C before and after 10 test cycles

**Samples**	**Scattering Pair**	**CN**	**R (Å)**	**σ^2(10^{-3}Å^2)**	**ΔE_0 (eV)**
Mn-O-C (As synthesised)	Mn-O	4.00	2.17	3.53	-1.02
Mn-O-C (After 10 test cycles)	Mn-O	4.00	2.20	5.64	1.38

CN is the coordination number; R is interatomic distance (the bond length between central atoms and surrounding coordination atoms); σ^2 is Debye-Waller factor (a measure of thermal and static disorder); ΔE_0 is edge-energy shift (the difference between the zero kinetic energy value of the sample and that of the theoretical model).

Table S3 Comparsion of NitRR performance between Mn-O-C catalyst and recently-reported electrocatalysts

**Eelectrolysts**	**NO_3^- concentration**	**Electrolyte**	**R_{NH_3} (μg h^{-1} cm^{-2})**	**FE (%)**	**Refs.**
PTCDA/O-Cu	500 ppm	0.1 M PBS	436 ± 85	77 ± 3 (−0.4 V *vs.* RHE)	[S5]
TiO_{2-x} nanotubes	50 ppm	0.5 M Na_2SO_4	~765	85 (−1.6 V *vs.* SCE)	[S6]
Cu/Cu_2O nanowire arrays	200 ppm	0.5 M Na_2SO_4	4163.3	95.8 (−0.85 V *vs.* RHE)	[S7]
CoO_x	0.1 M	0.1 M KOH	~2844 ± 168.9	93.4 ± 3.8 (−0.3 V *vs.* RHE)	[S8]
Fe-PPy SACs	0.1 M	0.1 M KOH	2750	~100 (−0.3 V *vs.* RHE)	[S9]
Fe SAC	0.5 M	0.1 M K_2SO_4	7820	~75 (−0.66 V *vs.* RHE)	[S10]
CoP NAs/CFC	1.0 M	1.0 M NaOH	16252	~100 (−0.3 V *vs.* RHE)	[S11]
Co-SACs	100 ppm	0.02 M Na_2SO_4	408	~92 (−0.69 V *vs.* RHE)	[S12]
Pd-NDs/Zr-MOF	500 ppm	0.1 M Na_2SO_4	1953.3	58.1 (−1.3 V *vs.* RHE)	[S13]
a-RuO_2	200 ppm	0.5 M Na_2SO_4	1968.6	97.46 (−0.35 V *vs.* RHE)	[S14]
Cu-N-CSAC	0.1 M	0.1 M KOH	4491.4	84.7 (−1.0 V *vs.* RHE)	[S15]
$BiFeO_3$	0.1 M	0.1 M KOH	5178.2	96.85 (−0.6 V *vs.* RHE)	[S16]

Cu@C	1 mM	1.0 M KOH	469.5	72.0 (−0.3 V *vs.* RHE)	[S17]
Cu-Ni tandem catalyst	0.1 M	0.01 M KOH + 0.5 M Na_2SO_4	9921.2 ± 23.8	88.0 ± 1.6 (−1.0 V *vs.* RHE)	[S18]
CuN_4&Cu_4	50 ppm	0.5 M Na_2SO_4	749.7	~94.3 (−0.75 V *vs.* RHE)	[S19]
Cu_xCo_yHHTP	0.1 M	0.5 M Na_2SO_4	5098.3	96.4 (−0.6 V *vs.* RHE)	[S20]
Mn-O-C	1000 ppm	0.1 M K_2SO_4	1476.9 ± 62.6	89.0 ± 3.8 (−0.5 V *vs.* RHE)	This work

Supplementary References

[S1] P. E. Blöchl, Projector augmented-wave method. Phys. Rev. B **50** (24), 17953–17979 (1994). https://doi.org/10.1103/PhysRevB.50.17953

[S2] G. Kresse, D. Joubert, From ultrasoft pseudopotentials to the projector augmented-wave method. Phys. Rev. B **59** (3), 1758–1775 (1999). https://doi.org/10.1103/PhysRevB.59.1758

[S3] J. P. Perdew, K. Burke, M. Ernzerhof, Generalized gradient approximation made simple. Phys. Rev. Lett. **78** (7), 1396–1396 (1997). https://doi.org/10.1103/PhysRevLett.77.3865

[S4] J. P. Perdew, J. A. Chevary, S. H. Vosko, K. A. Jackson, M. R. Pederson et al., Atoms, molecules, solids, and surfaces: Applications of the generalized gradient approximation for exchange and correlation. Phys. Rev. B **46** (11), 6671–6687 (1992). https://doi.org/10.1103/PhysRevB.46.6671

[S5] G.F. Chen, Y.F. Yuan, H.F. Jiang, S.Y. Ren, L.X. Ding et al., Electrochemical reduction of nitrate to ammonia via direct eight-electron transfer using a copper–molecular solid catalyst. Nat. Energy **5**, 605–613 (2020). https://doi.org/10.1038/s41560-020-0654-1

[S6] R.R. Jia, Y.T. Wang, C.H. Wang, Y.F. Ling, Y.F. Yu et al., Boosting selective nitrate electroreduction to ammonia by constructing oxygen vacancies in TiO_2. ACS. Catal. **10**(6), 3533–3540 (2020). https://doi.org/10.1021/acscatal.9b05260

[S7] Y.T. Wang, W. Zhou, R.R. Jia, Y.F. Yu, B. Zhang et al., Unveiling the activity origin of a copper-based electrocatalyst for selective nitrate reduction to ammonia. Angew. Chem. Int. Ed. **59**, 5350 (2020). https://doi.org/10.1002/anie.201915992

[S8] J. Wang, C. Cai, Y. Wang, X.M. Yang, D.J. Wu et al., Electrocatalytic reduction of nitrate to ammonia on low-cost ultrathin CoO_x nanosheets. ACS Catal. **11**, 15135–15140 (2021). https://doi.org/10.1021/acscatal.1c03918

[S9] P.P. Li, Z.Y. Jin, Z.W. Fang, G.H. Yu et al., A single-site iron catalyst with preoccupied acive centers that achieves selective ammonia electrosynthesis from nitrate. Energy Environ. Sci. **14**, 3522–3531 (2021). https://doi.org/10.1039/D1EE00545F

[S10] Z.Y. Wu, M. Karamad, X. Yong, Q.Z. Huang, D. A. Cullen et al., Eelectrochemical ammonia synthesis via nitrate reduction on Fe single atom catalyst. Nat. Commun. **12**, 2870 (2021). https://doi.org/10.1038/s41467-021-23115-x

[S11] S.H. Ye, Z.D. Chen, G.K. Zhang, W.D. Chen, C. Pen et al., Elucidating the activity, mechanism and application of selective electrosynthesis of ammonia from nitrate on cobalt phosphide. Energy Environ. Sci. **15**, 760–770 (2022). https://doi.org/10.1039/D1EE03097C

[S12] J.C. Li, M. Li, N. An, S. Zhang, Q.N. Song et al., Boosted ammonium production by single cobalt atom catalysts with high Faradic efficiencies. Proc. Natl. Acad. Sci. U.S.A. **119**, e2123450119 (2022). https://doi.org/10.1073/pnas.2123450119

[S13] M.H. Jiang, J. Su, X.M. Song, P.B Zhang, M.F. Zhu et al., Interfacial reduction nucleation of noble metal nanodots on redox-active metal–organic frameworks for high-efficiency electrocatalytic conversion of nitrate to ammonia. Nano Lett. **22**(6), 2529–2537 (2022). https://doi.org/10.1021/acs.nanolett.2c00446

[S14] Y.T. Wang, H.J. Li, W. Zhou, X. Zhang, B. Zhang et al., Structurally disordered RuO_2 nanosheets with rich oxygen vacancies for enhanced nitrate electroreduction to ammonia. Angew. Chem. Int. Ed. **61**, e202202604 (2022). https://doi.org/10.1002/anie.202202604

[S15] J. Yang, H.F. Qi, A.Q. Li, X.Y. Liu, X.F. Yang et al., Potential-driven restructuring of Cu single atoms to nanoparticles for boosting the electrochemical reduction of nitrate to ammonia. J. Am. Chem. Soc. **144**(27), 12062–12071 (2022). https://doi.org/10.1021/jacs.2c02262

[S16] J. Wang, D.J. Wu, M.H. Li, X.B. Wei, X.M. Yang et al., Bismuth ferrite as an electrocatalyst for the electrochemical nitrate reduction. Nano Lett. **22**(13), 5600–5606 (2022). https://doi.org/10.1021/acs.nanolett.2c02026

[S17] Z.M. Song, Y. Liu, Y.Z. Zhong, Q. Guo, J. Zeng et al., Efficient electroreduction of nitrate into ammonia at ultralow concentrations via an enrichment effect. Adv. Mater. **34**, 2204306 (2022). https://doi.org/10.1002/adma.202204306

[S18] Y.G. Bu, C. Wang, W.K. Zhang, X. Yang, J. Ding et al., Electrical pulse-driven periodic self-repair of Cu-Ni tandem catalyst for efficient ammonia synthesis from nitrate. Angew. Chem. Int. Ed. **62**, e202217337 (2023). https://doi.org/10.1002/ange.202217337

[S19] X.Y. J, K. Sun, Z.K. Liu, X.H. Liu, W.K. Dong et al., Identification of dynamic active sites among Cu species derived from MOFs@CuPc for electrocatalytic nitrate reduction reaction to ammonia. Nano-Micro Lett. **15**, 110 (2023). https://doi.org/10.1007/s40820-023-01091-9

[S20] P. Liu, J.Y. Yan, H. Huang, W.B. Song, Cu/Co bimetallic conductive MOFs: Electronic modulation for enhanced nitrate reduction to ammonia. Chem. Eng. J. **466**, 143134 (2023). https://doi.org/10.1016/j.cej.2023.143134
